# Physiological mechanisms determining eccrine sweat composition

**DOI:** 10.1007/s00421-020-04323-7

**Published:** 2020-03-02

**Authors:** Lindsay B. Baker, Anthony S. Wolfe

**Affiliations:** 1 Gatorade Sports Science Institute, PepsiCo R&D Life Sciences, 617 W. Main St., Barrington, IL 60010 USA; 2 Gatorade Sports Science Institute, PepsiCo R&D Life Sciences, Frisco, TX USA

**Keywords:** Glucose, Lactate, Ammonia, Urea, Bicarbonate, Amino acids, Ethanol, Cytokines, Electrolytes, Biomarker

## Abstract

**Purpose:**

The purpose of this paper is to review the physiological mechanisms determining eccrine sweat composition to assess the utility of sweat as a proxy for blood or as a potential biomarker of human health or nutritional/physiological status.

**Methods:**

This narrative review includes the major sweat electrolytes (sodium, chloride, and potassium), other micronutrients (e.g., calcium, magnesium, iron, copper, zinc, vitamins), metabolites (e.g., glucose, lactate, ammonia, urea, bicarbonate, amino acids, ethanol), and other compounds (e.g., cytokines and cortisol).

**Results:**

Ion membrane transport mechanisms for sodium and chloride are well established, but the mechanisms of secretion and/or reabsorption for most other sweat solutes are still equivocal. Correlations between sweat and blood have not been established for most constituents, with perhaps the exception of ethanol. With respect to sweat diagnostics, it is well accepted that elevated sweat sodium and chloride is a useful screening tool for cystic fibrosis. However, sweat electrolyte concentrations are not predictive of hydration status or sweating rate. Sweat metabolite concentrations are not a reliable biomarker for exercise intensity or other physiological stressors. To date, glucose, cytokine, and cortisol research is too limited to suggest that sweat is a useful surrogate for blood.

**Conclusion:**

Final sweat composition is not only influenced by extracellular solute concentrations, but also mechanisms of secretion and/or reabsorption, sweat flow rate, byproducts of sweat gland metabolism, skin surface contamination, and sebum secretions, among other factors related to methodology. Future research that accounts for these confounding factors is needed to address the existing gaps in the literature.

**Electronic supplementary material:**

The online version of this article (10.1007/s00421-020-04323-7) contains supplementary material, which is available to authorized users.

## Introduction

There has been considerable interest recently in sweat diagnostics, that is, the use of sweat as a non-invasive alternative to blood analysis to provide insights into human physiology, health, and performance. Since extracellular fluid is the precursor to primary sweat in the secretory coil of eccrine sweat glands (Cage and Dobson 1965; Sato 1977), it is then anticipated that sweat solute concentrations excreted onto the sweat surface provide a surrogate for blood or are at least directly correlated with that of blood. However, eccrine sweat is a very complex mixture of solutes (Sato 1977, 1993; Sato et al. 1989), of which the composition can change significantly along its passage in the sweat duct and during collection on the skin surface. Furthermore, there are still many unanswered questions about the mechanisms of sweat secretion and sweat composition. For instance, while ion membrane transport mechanisms for sodium (Na^+^) and chloride (Cl^−^) have been described (Quinton 1981; Sato 1993; Sato et al. 1991), the mechanisms underlying the concentrations of many other solutes in final sweat are poorly understood. Moreover, relatively few well-designed, adequately powered studies have investigated the correlation between sweat and blood solute concentrations. Thus, while the notion of sweat diagnostics as a non-invasive tool for real-time hydration, nutrition, and health monitoring is attractive, its application has been limited to date (Brothers et al. 2019; Gao et al. 2018; Heikenfeld et al. 2018).

Most publications on sweat diagnostics have primarily focused on the development of skin-interfaced platforms capable of capturing and performing quantitative measurements of sweat chemistry. These papers, mostly published in journals with a focus on engineering and sensor technology, have demonstrated significant advances in materials, mechanics, and microsystem designs (Bandodkar et al. 2019; Brothers et al. 2019; Salim and Lim 2019). Notwithstanding these technological advances, very few sensor studies discuss the composition of sweat from a physiological perspective or provide direct evidence from a mechanistic basis that the sweat solute concentrations measured at the surface of the skin provide insight into the participants’ hydration or nutritional status or other physiological changes.

To understand the potential utility of sweat as a reflection of blood, one must first have a thorough understanding of the physiological mechanisms determining the composition of sweat—from the composition of primary sweat in the secretory coil, to the mechanisms of reabsorption and secretion in the duct, which influence final sweat excreted onto the skin surface. While several excellent classic reviews on sweat composition and mechanisms of sweat gland function have been published (Costill 1977; Robinson and Robinson 1954; Sato 1977; Sato et al. 1989), very few recent reviews have provided an update on mechanisms underlying sweat composition in the context of sweat as a potential biomarker of human health (Baker 2019). It is critical to identify what is known and what is not yet known regarding the physiology of sweat composition to generate mechanism-based hypotheses and guide the direction of future research in the area of sweat diagnostics.

The objective of this paper is to review the physiological mechanisms determining eccrine sweat composition during passive and active heat stress. The review will include the major sweat electrolytes (Na^+^, Cl^−^, and potassium (K^+^)), as well as other micronutrients (e.g., calcium (Ca^2+^), magnesium (Mg^2+^), iron (Fe^2+^), copper (Cu^2+^), zinc (Zn^2+^), and vitamins), metabolites (e.g., glucose, lactate, ammonia, urea, bicarbonate, amino acids, and ethanol), and other compounds (e.g., cytokines and cortisol) present in sweat. The findings could help elucidate which sweat constituents, if any, could serve as a potential surrogate for blood, identify gaps in the literature, and inform the direction of future research.

## Eccrine sweat glands and thermoregulatory sweating

Sweat glands are classified into three main types: eccrine, apocrine, and apoeccrine (Sato 1993; Sato et al. 1989). Eccrine sweat glands will be the focus of this review because they are the most numerous (2–4 million), distributed across most of the body surface area (Sato 1983, 1993) and are responsible for the highest volume of sweat production. Evaporation of sweat is the primary avenue of heat loss during exercise and/or passive heat stress. When water on the skin surface is converted to water vapor, 2426 J of heat per 1 g of evaporated sweat is removed from the body (Sawka et al. 2011; Wenger 1972). Whole body sweating rates vary considerably, depending upon the evaporative requirement for heat balance (Bain et al. 2011; Gagnon et al. 2013), but have been reported to be ~ 0.2 to 3 L/h across a wide range of individuals, activities, and environmental conditions (Barnes et al. 2019). Whole body sweating rate can be > 3.0 L/h (Armstrong et al. 1986; Barnes et al. 2019; Bergeron 2003; Godek et al. 2005, 2010; Palmer and Spriet 2008). For example, Alberto Salazar’s sweating rate was reported to be 3.7 L/h during the 1985 Olympic Marathon (23.9–27.8 °C). Sweating rates this high are not commonly observed (< 1% of athletes based on normative data from Barnes et al. 2019); but when they are, extreme circumstances related to environment conditions, workload (running pace), fitness/acclimatization, and/or large body mass are usually involved. The rate of sweat production over the whole body is a product of active sweat gland density and the secretion rate per gland. The initial response at the onset of sweating is a rapid increase in the recruitment of sweat glands, followed by a more gradual increase in sweat secretion per gland (Kondo et al. 1998, 2001; Kuno 1956; Randall 1946).

The anatomical structure of the eccrine sweat gland consists of two main functional components: a secretory coil and a duct, both made up of a simple tubular epithelium (Sato 1983). The secretory coil has three types of cells: clear, dark, and myoepithelial. Upon stimulation, clear cells of the secretory coil secrete primary sweat, which is nearly isotonic with blood plasma (Costill 1977; Sato 1977; Sato and Sato 1990). Briefly, secretion of sweat by the clear cells occurs primarily in response to increases in body temperature (Nadel 1979; Nadel et al. 1971; Sato 1993; Wingo et al. 2010), which is sensed by central and skin thermoreceptors. In turn, this information is processed by the preoptic area of the hypothalamus to initiate the sudomotor response-mediated predominately by sympathetic cholinergic stimulation (Sato 1993) and to a lesser extent (~ 10%) by adrenergic stimulation (Sato 1973). Eccrine glands also secrete sweat in response to non-thermal stimuli (separate from changes in skin or body temperature) associated with exercise, such as central command, muscle metabo-/mechanoreceptors, osmoreceptors, and possibly baroreceptors (Shibasaki and Crandall 2010). The dark cells of the secretory coil are distinguishable by the abundance of dark cell granules in the cytoplasm, but their function is poorly understood (Sato 1993; Sato et al. 1991). The myoepithelial cells provide structural support against the hydrostatic pressure generated in the gland during sweat production (Sato 1983). The eccrine duct has two cell layers: basal and luminal cells. The primary function of the duct is reabsorption of Na^+^ and Cl^−^ ions, most of which occurs in the proximal duct (Sato 1983), resulting in a hypotonic final sweat excreted onto the skin surface (Sato 1977, 1993).

Apocrine and apoeccrine glands differ from eccrine glands in that they are limited to certain body regions and, therefore, play a lesser role in the overall volume of sweat secretion (Montagna and Parakkal 1974a; Robertshaw 1983; Sato et al. 1987). However, their secretions are still of interest and have potential utility for use as biomarkers. Apocrine and apoeccrine gland secretions are also important to understand because they can impact the composition of eccrine sweat, depending upon the region of sample collection. Briefly, apocrine sweat glands become active after puberty. They are larger than eccrine glands and are found in the axilla, breasts, face, scalp, and perineum. Apocrine glands open into hair follicles and produce a viscous sweat containing lipids, proteins, sugars, and ammonia (Montagna and Parakkal 1974a; Robertshaw 1983). Apoeccrine sweat glands develop from eccrine glands located in the axillary region between ~ 8 and 14 years of age. They produce copious salt-water secretions which empty directly onto the skin surface (Sato et al. 1987; Sato and Sato 1987b). For more information on apocrine and apoeccrine glands, the reader is referred to other detailed reviews (Hu et al. 2018; Sato et al. 1989; Weiner and Hellmann 1960).

**Fig. 1:**
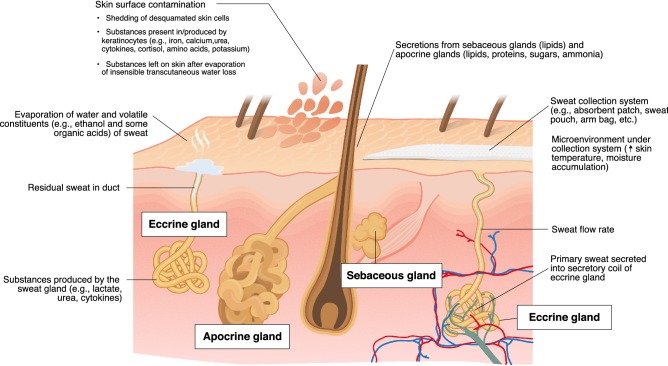
Physiological and methodological factors impacting the composition of final sweat collected from the skin surface

Figure 1 provides a general view of eccrine and apocrine sweat glands. For more details on thermoregulatory sweating and sweat gland structure/function, the reader is referred to more comprehensive reviews (Baker 2019; Gagnon and Crandall 2018; Sato 1983). Hereafter, the focus of the review will be sweat composition. Sweating rate will be discussed only in the context of its impact on sweat solute concentrations.

## Overview of sweat composition

Table 1 shows the range of concentrations of several constituents present in sweat. This is not intended to be an exhaustive list, but does include some of the more commonly researched substances in the sweat diagnostics literature. In the sections that follow, we will discuss the physiological mechanisms and other factors that determine the constituent concentrations measured in final sweat. Since eccrine sweat is mostly salt water, we will first discuss the electrolyte composition of sweat, focusing largely on the mechanisms of Na and Cl secretion and ductal reabsorption. As shown in Table 1, sweat contains a mixture of many other chemicals in varying concentrations, including additional micronutrients (e.g., K^+^, Ca^2+^, Mg^2+^, Fe^2+^, and vitamins) as well as metabolites (e.g., glucose, lactate, ammonia, urea, bicarbonate, amino acids, ethanol), cytokines, and cortisol. The concentrations listed in Table 1 are approximate ranges based on the available literature (see Supplemental Table 1 for references). Where possible, values were obtained from studies that took measures to prevent epidermal contamination (e.g., pre-rinsed skin, removed initial sweat, and/or analyzed cell-free sweat) in regional or whole body sweat. While normative data are available for Na^+^ and Cl^−^ (Barnes et al. 2019; Hammond et al. 1994; Wescor 2005), there are insufficient studies to identify reference ranges for other sweat constituents. However, one can see from Table 1 that Na^+^ and Cl^−^ are by far the most concentrated, ranging from ~ 10 up to ~ 90 mmol/L. Substances present at lower millimolar concentrations include lactate, ethanol, urea, ammonia, bicarbonate, and K^+^. Most other constituents shown in Table 1 are measured on a micromolar (Ca^2+^, Mg^2+^, Fe^2+^, Zn^2+^, Cu^2+^, ascorbic acid, glucose, and individual amino acids) or even smaller scale (nanomolar: thiamine, cortisol; picomolar: cytokines).

**Table 1:** Summary of select sweat constituents listed from highest to lowest concentrations

	Symbol	Sweat (mmol/L)	Blood^a^ (mmol/L)	Molecular weight (Da)	Other characteristics	Mechanism(s) of secretion in secretory coil	Mechanism(s) of ductal reabsorption
Sodium	Na	10–90	135–145	23	Ion ( +)	Paracellular transport	Epithelial Na channels and Na–K-ATPase
Chloride	Cl	10–90	98–107	35	Ion (−)	Na–K–2Cl cotransport model	CFTR
Lactate	C_3_H_5_O_3_	5–40	0.5–25	90	Polar molecule	Byproduct of sweat gland metabolism; transported out of clear cells via MCT proteins?	NA
Urea	CH_4_N_2_O	4–12	2.5–7.1	60	Polar molecule	Passive diffusion via transcellular or paracellular transport? Transcellular transport via UT-1?	NA
Potassium	K	2–8	3.6–5.2	39	Ion (+)	Na–K–2Cl cotransport model	NA
Ammonia	NH_3_	1–8	0.01–0.03	17	Polar molecule	Passive diffusion via transcellular or paracellular transport?	NA
Ethanol	C_2_H_5_OH	0–7	0–7	46	Polar molecule, with both hydrophilic and hydrophobic properties	Passive diffusion via transcellular or paracellular transport	NA
Bicarbonate	HCO_3_^−^	0.5–5	22–29	61	Polar molecule	HCO_3_/Cl channels (Best2) in the dark cells and HCO_3_/Cl exchanger in luminal cell?	CFTR combined with H secretion and HCO_3_/Cl exchanger in luminal cell?
Calcium	Ca	0.2–2	2.2–2.7	40	Ionized (++) and bound	Paracellular transport?	NA
Magnesium	Mg	0.02–0.40	0.7–0.95	24	Ionized (++) and bound	Paracellular transport?	NA
Glucose	C_6_H_12_O_6_	0.01–0.20	3.9–7.8	180	Polar molecule	Paracellular transport; Transcellular transport via GLUT2, SGLT3, SGLT4?	NA
Amino Acids	R–CH(NH_2_) –COOH	≤ 13 × 10^–3^ each	≤ 6.5 × 10^–3^ each	75–204 each	Polar and nonpolar	Leaching of natural moisturizing factors from the skin	NA
Iron	Fe	0.1–30 × 10^–3^	6–27 × 10^–3^	56	Mostly protein bound	Paracellular transport or transcellular carrier-mediated system?	NA
Copper	Cu	0.5–20 × 10^–3^	12–23 × 10^–3^	64	Mostly protein bound	Paracellular transport or transcellular carrier-mediated system?	NA
Zinc	Zn	0.1–20 × 10^–3^	10–17 × 10^–3^	65	Mostly protein bound	Paracellular transport or transcellular carrier-mediated system?	NA
Ascorbic Acid	C_6_H_8_O_6_	1–10 × 10^−3^	20–110 × 10^–3^	176	Polar molecule	Paracellular transport?	NA
Thiamine	C_12_H_17_N_4_OS^+^	≤ 13 × 10^–5^	7–18 × 10^–5^	266	Polar molecule	Paracellular transport?	NA
Cortisol	C_21_H_30_O_5_	0.06–0.70 × 10^–5^	5–70 × 10^–5^	362	Nonpolar molecule; mostly protein bound	Passive diffusion of unbound fraction only	NA
Cytokines	–	0.07–1.0 × 10^–9^ each	≤ 600 × 10^–9^ each	~ 16 to 25 kDa each	Polar molecules	Production by eccrine glands	NA

Supporting references for sweat concentrations are in Supplemental Table 1

^a^Blood values are normal reference ranges for adults obtained from Mayo Clinic Laboratories Test Catalog (https://www.mayocliniclabs.com/test-catalog/index.html). Specimen type varies among the constituents—reported ranges apply to serum (cortisol, urea, iron, calcium, sodium, potassium, chloride, bicarbonate, magnesium, ethanol, copper, zinc), plasma (cytokines, ammonia, lactate, amino acids, ascorbic acid), serum or plasma (glucose) or whole blood (thiamine). Range for plasma lactate concentration includes rest (0.5–2.2 mmol/L) and exercise (up to ~ 15 to 25 mmol/L in response to “all-out” maximal exertion) (Goodwin et al. 2007). NA, not applicable or no data available

For each constituent, reference ranges for corresponding blood plasma concentrations are also shown in Table 1. Because extracellular fluid is the precursor fluid for primary sweat, it follows that many components of the blood plasma are also found in final sweat. However, as shown in Fig. 1, there are many other factors impacting solute concentrations of final sweat collected from the skin surface. For example, some substances do not originate from precursor sweat (extracellular fluid), but instead enter the sweat gland as a result of production by the eccrine gland (e.g., lactate, urea, and cytokines) or appear in final sweat on the skin surface via contact with keratinocytes (e.g., iron, calcium, urea, cytokines, cortisol, amino acids, and potassium). There are also methodological factors that influence and often confound sweat composition, as will be discussed in detail in the next section.

## Overview of sweat collection and analytical techniques

### Collection methods

Scientists and practitioners have employed various methods to collect sweat samples for composition analysis. Whole body washdown is considered the most accurate method because sweat runoff from the entire body surface area is collected and accounted for and it does not interfere with normal evaporative sweating. Recovery of water and Na^+^ using this method has been reported to be ~ 99 to 102% and has a within-method coefficient of variation (CV) of ~ 4% (Baker et al. 2018b; Shirreffs and Maughan 1997). For these reasons, the WBW method is recommended, especially when conducting nutrient balance studies or if interested in determining total loss of micronutrients or other constituents via sweating. However, WBW is a meticulous method that requires a well-controlled laboratory setting. Therefore, in most studies, sweat is collected from one or more small regions of the body using a myriad of techniques, which have been described in detail elsewhere (Baker 2017, 2019; Taylor and Machado-Moreira 2013). Briefly, regional sweat collection techniques for sweat composition analysis include scraping or dripping methods, filter paper, absorbent patches, sweat pouches, sweat capsules, arm gloves/bags, and sweat collectors (e.g., Macroduct^®^/Megaduct). While regional sweat collection is practical, each method has its limitations, and some more than others. For example, covering the skin surface with the collection system can alter the local environment, impacting skin temperature and/or skin wettedness (Fig. 1). In turn, these changes can have confounding effects on the sweating process and/or introduce skin surface contaminants into the sweat sample. On the other hand, open-air collection techniques (scraping or dripping methods) allow evaporation of water and volatile constituents of sweat (Buono 1999; Talbert 1922) (Fig. 1). Depending on the collection device (material), the exact methods used (timing, skin preparation, etc.), and the constituent of interest, the confounding effect (background noise) could range from negligible to very large. These factors will be discussed in more detail in “Contamination considerations”.

Another important consideration is inter-regional variability in the sweat constituent concentrations. It is well documented that many sweat micronutrient (e.g., Na^+^, Cl^−^, Ca^2+^, Fe^2+^, Mg^2+^, Zn^2+^, Cu^2+^) concentrations can vary by up to two- to fourfold among anatomical sites (Aruoma et al. 1988; Baker et al. 2019, 2009; Patterson et al. 2000; Vellar and Askevold 1968). Inter-regional variability data are sparse for most other sweat constituents, but the few studies available seem to follow the same general trend, with sweat amino acid concentrations reported to vary by up to twofold (Liappis and Hungerland 1972), sweat lactate by up to two- to fourfold (Collins 1962; Patterson et al. 2000) and sweat bicarbonate by up to sixfold (Patterson et al. 2000) among body regions. One study measured the metabolomic profiles of sweat samples collected from the forearm, lower back, and neck (Hooton and Li 2017) over a 24-h period. The authors noted that the concentrations of many of the metabolites were very different among sites (shown via heat maps), but the inter-regional differences were not quantified.

Regional sweat concentrations can also differ significantly from WBW measurements. For example, sweat [Na^+^] and [Cl^−^] measured from the forehead, torso, and upper arms are often greater than whole body measurements. However, sweat [Na^+^] and [Cl^−^] from other sites such as the foot, calf, and thigh are similar to that of the whole body (Baker et al. 2019, 2009, 2018b; Patterson et al. 2000). For most other constituents (Ca^2+^, Mg^2+^, Fe^2+^, Zn^2+^, Cu^2+^, lactate, urea, ammonia, and lead), regional sweat measurements have led to overestimations of whole body sweat concentrations (Baker et al. 2011; Cohn and Emmett 1978; Colombani et al. 1997; Lemon et al. 1986; Patterson et al. 2000) albeit regional body mapping has not been as thorough (i.e., included as many regions) as that of Na^+^ and Cl^−^ studies. The physiological explanation underlying the inter-regional variability is unclear, but may be related to inter-regional differences in sweating rate and/or skin characteristics (e.g., presence of sebaceous glands, apocrine glands, hair/nails, etc.) as discussed in the next section.

### Contamination considerations

As summarized in Fig. 1, there are several confounding factors that play a role in determining sweat composition. For instance, if sweat is collected in an area dense with apocrine glands and/or sebaceous glands (e.g., axilla, face, forehead, scalp) their secretions could artificially elevate lipid, protein, sugar, and ammonia concentrations of sweat samples collected from the skin surface (Montagna and Parakkal 1974a, b; Porter 2001). Other substances present in sweat may be there as a result of production in the sweat gland rather than (or in addition to) having originated from primary sweat secretion into the secretory coil. Some substances potentially produced by the eccrine gland include lactate, urea, cytokines, proteolytic enzymes, and antibodies (Gordon et al. 1971; Murota et al. 2015; Sato 1977; Sato et al. 1991; Sato and Sato 1994; Yokozeki et al. 1991), but mechanisms are not fully understood.

Residual sweat in the eccrine duct left over from a previous thermal event could also confound sweat composition initially excreted on the skin surface (i.e., at the beginning of exercise or heat stress). Similarly, skin surface contamination could originate from minerals, amino acids, and other constituents deposited on the skin after evaporation of everyday insensible transcutaneous water loss (Mitchell and Hamilton 1949; Rothman et al. 1949). As shown in Supplemental Table 2, several studies have found significantly higher constituent concentrations in initial sweat samples versus subsequent collections (Boysen et al. 1984; Brune et al. 1986; Ely et al. 2011; Paulev et al. 1983). For example, Paulev et al. (1983) collected sweat from the back of endurance athletes at 10-min intervals during cycling and found that sweat [Fe^2+^] gradually decreased (by 2.2-fold) over the first 20–30 min and did not change thereafter. Similar results have been reported by other investigators for Fe^2+^, Ca^2+^, Mg^2+^, Cu^2+^, Zn^2+^, protein, and urea (Boysen et al. 1984; Brune et al. 1986; Ely et al. 2011). For these reasons, it is recommended that the skin is cleaned (e.g., alcohol wipe) and rinsed with deionized water and that initial sweat is removed from the skin surface prior to sweat collection. Allowing participants to sweat for at least 20–30 min prior to applying the sweat collection device will facilitate flushing of duct and surface contaminants and will allow time for steady-state sweating rates to be reached.

Scientists have also assessed the impact of skin surface contamination by comparing sweat collected with and without an oil barrier (Supplemental Table 2). The advantage of this method, also known as the anaerobic sweat pouch technique, is that the oil interface prevents contact between the skin and the sweat. Using this approach, one study found that urocanic acid concentration was sevenfold higher in arm sweat collected with the filter paper technique versus the anaerobic method (Brusilow and Ikai 1968). Boysen et al. (1984) found 2.5-fold higher [Ca^2+^], 1.6-fold higher [protein], and 1.4-fold higher [urea] in sweat collected from the upper back using the pouch technique without oil compared with the pouch with oil. Using the scraping method resulted in even more epidermal contamination, as Boysen et al. (1984) reported 6-fold, 2.7-fold, and 2.4-fold increases in sweat [Ca^2+^], [protein], and [urea], respectively, in scraped sweat versus the anaerobic method. Yet another approach to assessing the impact of epidermal contamination on sweat composition is to separate a sample into cell-rich and cell-poor aliquots. Cell-rich sweat is defined as the sweat analyzed ‘as is’ after collection from the skin surface, while cell-poor sweat is the supernatant obtained after centrifugation of that sample. Several studies (listed in Supplemental Table 2) employed this method when assessing sweat Fe and found 2- to 24-fold higher concentrations in cell-rich versus cell-poor sweat (Adams et al. 1950; Brune et al. 1986; Foy and Kondi 1957; Hussain and Patwardhan 1959; Prasad et al. 1963).

The potential for skin surface contamination to impact sweat sample concentrations varies depending upon the constituent of interest. Several substances that are of interest for sweat diagnostics are present in or produced by keratinocytes. As shown in Fig. 1, the skin-derived substances likely to confound sweat concentrations include (but are not limited to) Fe^2+^ (Weintraub et al. 1965), Ca^2+^ (Verissimo et al. 2007), K^+^ (Verissimo et al. 2007), cytokines (Dai et al. 2013; Hanel et al. 2013), cortisol (Slominski 2005; Terao and Katayama 2016), and amino acids (Dunstan et al. 2016; Sato et al. 1989). Using nuclear microscopy, Verissimo et al. (2007) obtained quantitative profiles of various elements in skin cross sections. It was found that some minerals, such as Ca^2+^, were abundant in the dead cells of the stratum corneum and less so in the living cells of the deeper layers of the epidermis. This is important because the content of the stratum corneum is likely to have the greatest impact on sweat sample contamination (through mixing of desquamated skin cells with sweat on the skin surface). According to Verissimo et al. (2007), there is ~ 500 to 2500 ng of Ca^2+^ present in a cm^2^ of the stratum corneum. If this amount of Ca^2+^ were incorporated into a 1 mL sample of sweat collected over a 10 cm^2^ surface area (typical absorbent patch size), it would equate to 0.12–0.62 mmol/L of Ca^2+^ contaminating the sweat [Ca^2+^] value. To put this in perspective, the range in sweat [Ca^2+^] typically reported is 0.2 to 2.0 mmol/L (Baker 2019). Based on these estimations, skin surface contamination could artificially elevate sweat [Ca^2+^] by two- to fourfold, which is in line with findings from studies comparing cell-rich vs. cell-poor sweat in Supplemental Table 2 (Boysen et al. 1984; Ely et al. 2011). By contrast, sweat [Na^+^] and [Cl^−^] seem to be minimally impacted by surface contamination (Arn and Reimer 1950; Boysen et al. 1984; Ely et al. 2011; Freyberg and Grant 1937; Robinson and Robinson 1954).

Taken together, it is apparent that the composition of sweat collected from the skin surface is influenced by not only internal sweat secretion mechanisms but also epidermal composition itself. To complicate matters further, the degree of contamination varies depending upon the method/duration of sweat collection and sweating rate. For example, prolonged encapsulation of the skin with occlusive coverings leads to moisture accumulation, which in turn accelerates desquamation. This may explain the very high concentrations of minerals often reported in studies using the arm bag technique (Cohn and Emmett 1978; Collins et al. 1971; Ely et al. 2011; Van Heyningen and Weiner 1952). In addition, for a given amount of mineral or metabolite from epidermal sources, the impact on measured sweat mineral concentration will be influenced by sweating rate such that higher sweat volumes will dilute the effect of epidermal contamination (Costill 1977; Dunstan et al. 2016). Finally, it is also important to consider potential environmental contamination, such as condensation of water vapor onto the skin surface in a sauna (Zech et al. 2015).

### Sample storage and analytical techniques

Sample storage methodology is another potential source of variability in sweat constituent concentrations and, not surprisingly, one of the most important factors is proper sealing of the sample vials. When well-sealed and stored at room temperature (23–25 °C), refrigeration (4–8 °C), or frozen (− 20 °C) for up to 7 days, minimal sweat evaporation and minimal change in sweat [Na^2+^], [Cl^−^], and [K^+^] has been reported (Baker et al. 2018a; Bergeron et al. 2011; Jones et al. 2008). However, when vials were capped but not sealed with Parafilm-M^®^ for 3 or 5 days, 19% and 32% sample evaporation has occurred; storing the vials in a plastic bag was associated with 16 and 27% sample evaporation, respectively. This translates to a significant increase in sweat electrolyte concentrations. For example, Bergeron et al. (2011) reported a 12–66% increase in sweat [Cl^−^] after 5 days of storage without Parafilm-M^®^ sealing.

Differences in sample storage temperature seem to have minimal impact on sweat electrolyte concentrations, as (Baker et al. 2018a) found negligible changes in sweat [Na^+^], [Cl^−^], and [K^+^] between same-day analysis and post-7-day storage (in Parafilm-M^®^-sealed vials) at − 20 °C, 8 °C, and 23 °C. There is a paucity of research measuring the effect of storage conditions on other sweat constituents. One study compared amino acid concentrations in aliquots of sweat stored at room temperature (25 °C) versus 37 °C for up to 90 min, prior to longer term storage at − 80 °C (Harshman et al. 2019). The authors reported that, in general, amino acid concentrations were stable at both conditions, with 90% of measurements being within 10% of the control condition (flash freezing). However, within certain individuals there were large differences in sweat alanine, arginine, and threonine concentrations between storage conditions (Harshman et al. 2019).

Because of differences in ease of use and equipment cost, a wide range of analytical techniques have been used by scientists and practitioners to measure sweat composition (Baker 2017). However, caution should be used when comparing results across the literature because sweat [Na^+^] varies significantly among common analytical techniques; in general, ion conductivity > flame photometry ≥ indirect ion-selective electrode > direct ion-selective electrode ≥ ion chromatography (Baker et al. 2014; Boisvert and Candas 1994; Dziedzic et al. 2014; Goulet et al. 2017, 2012). One study directly compared sweat [Na^+^] analyzed with all five different techniques (Goulet et al. 2017). While all techniques had low within-method variability (CVs ≤ 2.6%), there was significant variability between techniques, with ion conductivity producing sweat [Na^+^] values most different (CV = 12.3%) from the reference ion chromatography method (Goulet et al. 2017). Sweat [Cl^−^] and [K^+^] have been assessed in only a few studies, but follow a similar pattern as [Na^+^] with respect to differences among analytical techniques (Baker et al. 2014; Boisvert and Candas 1994). Future research is needed to determine the effect of variations in storage temperature/duration and analytical technique on other sweat constituents.

## Mechanisms determining sweat composition

The precursor fluid to primary sweat is the water and dissolved solutes in the interstitial fluid space, which together with the plasma makes up the extracellular fluid (in approximate equilibrium). There are two basic mechanisms by which water and solutes can cross the lipid bilayer membrane of clear cells and into the lumen of the secretory coil: transcellularly (through cells) or paracellularly (between cells) (Kutchai 1998). Passive diffusion via the transcellular and paracellular route is governed primarily by polarity and size of the constituent of interest (Yang and Hinner 2015). Nonpolar lipid-soluble molecules and small gasses like O_2_ diffuse rapidly with permeability coefficients reported as high as 2.3 × 10^1^ cm/s (Yang and Hinner 2015). Small polar molecules such as ammonia exhibit a relatively high permeability coefficient on the order of 10^–1^ cm/s, whereas urea, a slightly larger polar molecule, is closer to 10^–6^ cm/s (Walter and Gutknecht 1986; Yang and Hinner 2015). Transcellular permeability is nearly non-existent for large uncharged polar molecules like glucose. Passive movement of water and solutes occurs according to electrochemical gradients. Paracellular movement of solutes against concentration gradients can also occur with the help of ion transporters (e.g., Na^+^–K^+^-ATPase) via active or energy-requiring processes (Yang and Hinner 2015). Larger polar molecules and charged ions or molecules move paracellularly during the process of sweat secretion (Heikenfeld et al. 2019). Paracellular permeability is dictated by tight junctions, which allow free passage of water and small polar molecules but limit the passage of larger molecules (Kutchai 1998). However, tight junctions are dynamic and their bond strength varies depending on the cell type and when exposed to stressors (Schneeberger and Lynch 1992).

**Fig. 2:**
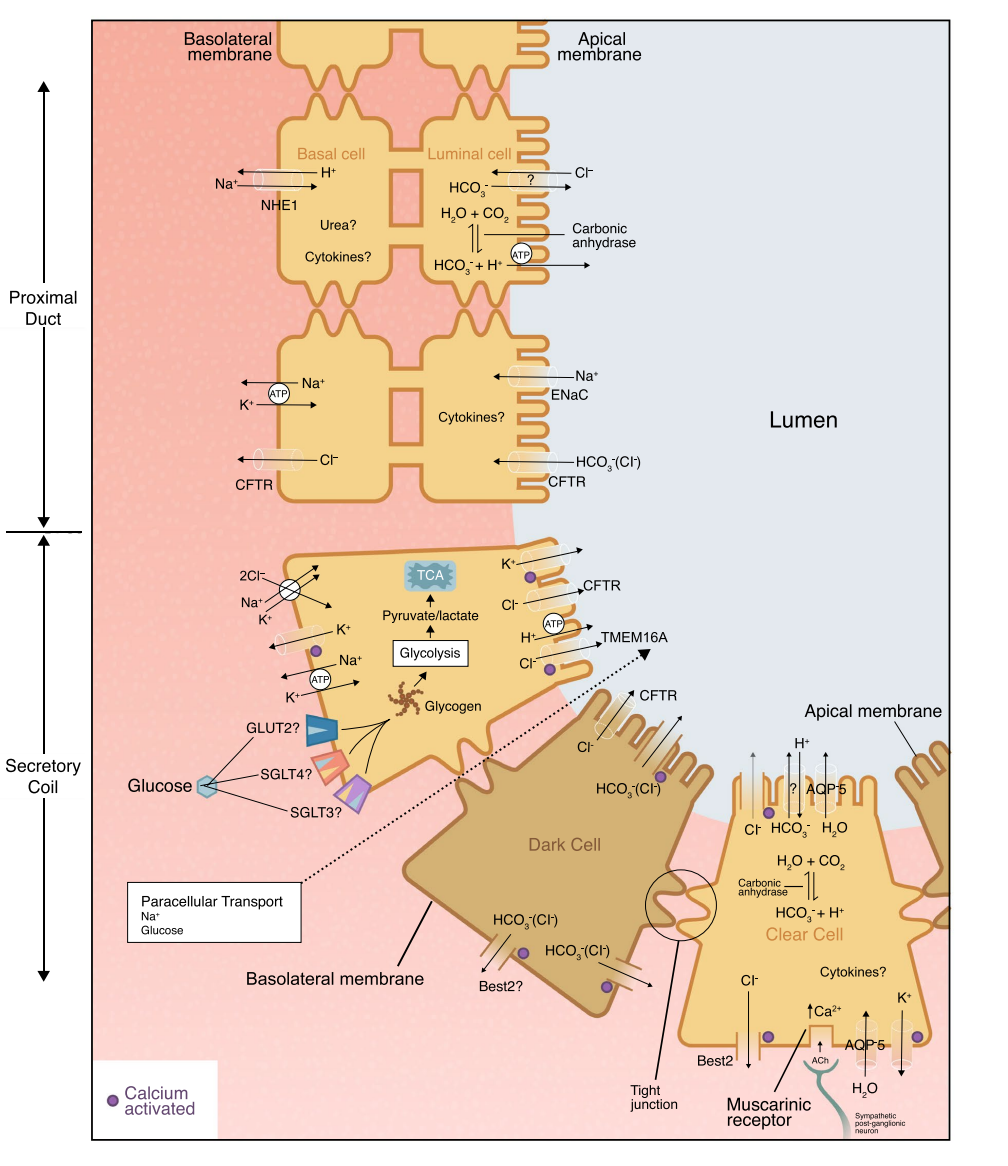
Mechanisms of water, ion, and metabolite passage into the secretory coil. *ACh* acetylcholine, *AQP-5* aquaporin-5, *Best2* bestrophin 2, *CFTR* cystic fibrosis transmembrane conductance regulator (note that chloride secretion via CFTR in the clear cells is activated by beta-adrenergic stimulation, which is not depicted), *ENaC* epithelial Na channel, *GLUT2* glucose transporter 2, *NHE1* Na^+^/H^+^ exchanger isoform 1, *SGLT3* sodium-dependent glucose transporter 3, *SGLT4* sodium-dependent glucose transporter 4, *TMEM16A* transmembrane member 16A

Mechanisms of water and electrolyte (Na^+^, Cl^−^, and K^+^) secretion and reabsorption in the eccrine sweat gland have been fairly well studied (Buono et al. 2007; Cage and Dobson 1965; Gerrett et al. 2019; Reddy and Quinton 2003; Sato 1983, 1993). Thus, current knowledge (as well as some remaining gaps in knowledge) of the transporters/channels, their regulatory control, and other mechanisms involved in Na^+^, Cl^−^, and K^+^ movement across cell membranes in the eccrine gland are discussed in detail below. On the other hand, the exact mechanisms determining the secretion of other ions or compounds in the secretory coil are largely unknown. Based on the available literature and basic concepts in membrane physiology, some potential mechanisms will be discussed for these solutes. Figure 2 shows the mechanisms of water and solute passage into the secretory coil.

### Electrolytes (sodium, chloride, and potassium)

Na^+^ and its conjugate anion Cl^−^ comprise the most osmotically active components of the extracellular fluid. K^+^ is the major cation in the intracellular fluid compartment. Electrolyte balance plays an important role in governing passive water movement according to osmotic gradients between the intracellular and extracellular water spaces (Mack and Nadel 1996). Because Na^+^ is the primary extracellular osmolyte, ingestion of Na^+^ helps maintain extracellular fluid volume, including plasma volume. Furthermore, an increase serum [Na^+^] and osmolality with Na^+^ ingestion stimulates renal water reabsorption for more complete rehydration (Evans et al. 2017). Individuals with salty sweat (e.g., [Cl^−^] and [Na^+^] ≥ 70 to 80 mmol/L) have an increased risk of NaCl imbalances during prolonged periods of heavy sweating (Baker 2019; Montain et al. 2006). Because of the large inter-individual variability in sweating rate and sweat [Na^+^] personalized fluid/Na^+^ replacement strategies are recommended to maintain fluid and Na^+^ balance during exercise (Belval et al. 2019; McDermott et al. 2017; Sawka et al. 2007).

#### Secretory mechanisms

Secretion of primary sweat occurs in clear cells of the secretory coil according to the Na^+^–K^+^–2Cl^−^ cotransport model (Hu et al. 2018; Sato 1993; Sato et al. 1991; Wilson and Metzler-Wilson 2015) illustrated in Fig. 2. Briefly, binding of acetylcholine to muscarinic receptors on the clear cell stimulates the release of intracellular Ca^2+^ from the sarcoplasmic reticulum (via IP_3_-activated Ca^2+^ and Ca^2+^-induced Ca^2+^ release channels, not depicted in Fig. 2) and an influx of extracellular Ca^2+^ into the cytoplasm (via TRPV1, Orai, TRPC, and L-type voltage-gated Ca^2+^ channels, not depicted in Fig. 2). An efflux of K^+^ (via Ca^+^ activated IK and BK channels) and Cl^−^ (via TMEM16A and Best2) then leads to cell shrinkage, triggering an influx of Na^+^, K^+^, and Cl^−^ from the extracellular fluid through Na^+^–K^+^–2Cl^−^ cotransporters on the basolateral membrane. Subsequently, Na^+^ and K^+^ efflux occurs through Na^+^–K^+^-ATPase and K^+^ channels (IK and BK) on the basolateral membrane as well as Cl^−^ efflux into the lumen via Cl^−^ channels (TMEM16A and Best2) on the apical membrane. CFTR are also expressed on the apical membrane of clear cells and play a role in chloride secretion in response to beta-adrenergic stimulation (Saint-Criq and Gray 2017). An electrochemical gradient is created by increased Cl^−^ concentration in the lumen. In turn, Na^+^ secretion occurs through passive movement across the cell junction (paracellular transport) (Sato 1993; Sato et al. 1989, 1991). The net K^+^ and Cl^−^ efflux also creates an osmotic gradient for water movement into the lumen via aquaporin-5 channels (Inoue et al. 2013; Nejsum et al. 2002; Xie et al. 2017). Thus water, Na^+^, Cl^−^, and K^+^ are secreted via this Na^+^–K^+^–2Cl^−^ cotransport model (Fig. 2) and primary sweat in the lumen of the secretory coil is nearly isotonic with blood plasma with respect to Na^+^ and K^+^ and slightly hypertonic for Cl^−^ (Dobson 1965; Sato 1977; Sato and Sato 1987a, 1990).

#### Reabsorption mechanisms

It is important to note, however, that the electrolyte concentrations of final sweat usually differ (i.e., hypotonic with respect to Na^+^ and Cl^−^) from that of primary sweat. This is a result of Na^+^ and Cl^−^ reabsorption in the eccrine duct prior to sweat excretion onto the skin surface. Reabsorption of Na^+^ and Cl^−^ is thought to occur via the modified Ussing leak-pump model (Sato 1977; Ussing and Zerahn 1951). As shown in Fig. 2, the mechanism involves passive influx of Na^+^ through amiloride-sensitive epithelial Na^+^ channels in the apical membrane of the luminal cells. This is followed by active transport of Na^+^ (via Na^+^–K^+^-ATPase) across the basolateral membrane of the basal cells and passive efflux of K^+^ through the basolateral membrane. Cl^−^ reabsorption is largely passive via movement through cystic fibrosis transmembrane conductance regulator (CFTR) channels on both the apical and basolateral membranes (Quinton 1981; Reddy and Quinton 1994b; Sato 1993). Most Na^+^ and Cl^−^ reabsorption occurs in the proximal segment of the eccrine duct, as these cells contain more mitochondria and Na^+^–K^+^-ATPase activity than that of the distal duct (Sato 1983). Sweat secretion and Na^+^ reabsorption are active processes and the main avenue of energy production for sweat gland function is oxidative phosphorylation of plasma glucose (Dobson and Sato 1972; Elizondo 1973; Elizondo et al. 1972; Sato 1977; Sato and Dobson 1973; Weiner and Van Heyningen 1952). Sweat gland metabolism and its byproducts can have an impact on final sweat composition, as discussed further in “Metabolites”. In addition, impeding oxygen supply to the sweat gland inhibits sweat secretion and ion reabsorption, resulting in reduced sweating rates and increased lactate and NaCl concentrations (Elizondo 1973; Elizondo et al. 1972; Sato 1977; Weiner and Van Heyningen 1952).

Taken together, the [Na^+^] and [Cl^−^] of final sweat are determined primarily by the rate of ion reabsorption in the duct relative to the rate of ion secretion in the clear cells of the secretory coil. Therefore, Na^+^–K^+^-ATPase activity and CFTR channel function play important roles in determining final sweat [Na^+^] and [Cl^−^]. The activity of Na^+^–K^+^-ATPase is influenced by plasma aldosterone concentration and sweat gland sensitivity to aldosterone (Kirby and Convertino 1986; Ladell and Shephard 1961; Sato and Dobson 1970). Resting (genomic) plasma aldosterone concentration is influenced by an individual’s chronic physiological condition (e.g., heat acclimation, fitness, and diet). Non-genomic factors (e.g., exercise and dehydration) stimulate acute changes in circulating aldosterone (Yoshida et al. 2006). The relative importance of these genomic and non-genomic factors on final sweat [Na^+^] will be covered in more detail below.

The abundance of CFTR Cl^−^ channels also impacts the rate of ductal [Na^+^] and [Cl^−^] reabsorption. With lower CFTR abundance, there is less ion reabsorption in the duct and, therefore, higher final sweat [Na^+^] and [Cl^−^] excreted onto the skin surface. The reduced reabsorption of Na^+^ as well as Cl^−^ is a result of the functional interaction between CFTR and ENaC, as ENaC activation depends on functioning CFTR (Reddy and Quinton 2003). CFTR availability is reduced with defects in CFTR genes (i.e., cystic fibrosis) (Goodman and Percy 2005; Quinton 1999, 2007; Reddy and Quinton 2003; Rowe et al. 2005). Thus, with cystic fibrosis, defects in CFTR also impose a loss of ENaC activity (Reddy and Quinton 2003). There is also some evidence to suggest that healthy individuals with salty sweat may exhibit lower abundance of eccrine duct Cl^−^ channel CFTR (Brown et al. 2011).

##### Sweat flow rate

Sweat flow rate is another important factor determining final sweat [Na^+^] and [Cl^−^]. Several studies have reported a direct relation between sweating rate and final sweat [Na^+^] and [Cl^−^] (Buono et al. 2007; 2008; Cage and Dobson 1965; Dill et al. 1938; Lichton 1957; Sato 1977,1983; Sato et al. 1989). This finding is consistent whether measured at the whole-body level (Baker et al. 2019), within given skin regions (Buono et al. 2008; Cage and Dobson 1965), or isolated sweat glands (Sato 1977). For example, Buono et al. (2008) found that as forearm sweating rate increased from ~ 0.25 to 0.82 mg/cm^2^/min (stimulated via a progressive increase in exercise intensity from 50 to 90% HR_max_), sweat [Na^+^] increased from 19 ± 5 to 59 ± 10 mmol/L. This is because the rate of Na^+^ secretion in primary sweat increased proportionally more than the rate of Na^+^ reabsorption in the duct (Buono et al. 2008). That is, the percentage of secreted Na^+^ that was reabsorbed in the duct decreased with a rise in sweating rate. Specifically, 86 ± 3% of the secreted Na^+^ was reabsorbed at the lowest sweating rate, while only 65 ± 6% of Na^+^ was reabsorbed at the highest sweating rate (Buono et al. 2008). Further research is needed to elucidate underlying mechanisms to explain why faster flow rates are associated with a decreased percentage of Na^+^ reabsorption.

It is important to note that while the relation between sweat flow rate and sweat [Na^+^] has been well established in studies where participants served as their own control, this relation does not necessarily hold true for between-participant comparisons (Baker et al. 2018b). It may be that other factors affecting sweat [Na^+^] and [Cl^−^], such as CFTR abundance in the eccrine duct or genomic effects of aldosterone on Na^+^–K^+^-ATPase, play a larger role (than acute changes in sweating rate) in determining inter-individual differences in sweat [Na^+^] and [Cl]. This complexity makes it all the more difficult to use sweat [Na^+^] to estimate sweating rate or other biomarkers.

##### Skin temperature

While it is well established that increases in skin temperature enhance sweat production (Nadel et al. 1971), the effect of skin temperature on sweat composition is less clear. Some (Johnson et al. 1944; Robinson et al. 1950; Weiner and Van Heyningen 1952), but not all (Bulmer and Forwell 1956), early studies found that higher skin temperatures were associated with higher sweat [Na^+^] and [Cl^−^]. More recently, Shamsuddin et al. (2005) investigated the effect of changes in mean skin temperature on ion reabsorption rates as measured via assessments of the relation between sweating rate and sweat conductivity. Moderately warm ambient conditions (25 °C) resulted in a lower threshold and greater slope of the relation between local (back) sweating rate and sweat conductivity compared with cool conditions (15 °C) (Shamsuddin et al. 2005). Based on these results, Shamsuddin et al. (2005) suggested that ion reabsorptive capacity of eccrine glands was increased by higher (by ~ 3 °C) mean skin temperatures. Indeed, some in vitro experiments suggest that ion channel (ENaC) activity is temperature dependent, such that higher temperature increases excitability and open probability (Chraibi and Horisberger 2002, 2003; Ruff 1999).

It is important to note, however, that sweating rate and sweat ion conductivity (index of sweat ion concentration) in the study by Shamsuddin et al. (2005) were actually higher in the 25 °C than the 15 °C trial. This agrees with the notion (discussed in “Sweat flow rate”) that final sweat [Na^+^] and [Cl^−^] increase with increasing sweating rate. However, there may be an interaction effect, such that changes in skin temperature impact the relation between sweating rate and ion concentration. That is, at a given sweating rate, sweat ion concentration may be lower with warmer skin temperatures because of enhanced reabsorptive capacity in the sweat duct (Shamsuddin et al. 2005). Nevertheless, research to date indicate that relatively large increases in mean (> 3 °C) or local (> 6 °C) skin temperature (Gerrett et al. 2019) are required before ion reabsorption rates are significantly affected.

#### Final sweat sodium, chloride, and potassium concentrations

The mechanisms of Na^+^ and Cl^−^ secretion and reabsorption along with the various modifying factors described above illustrate why sweat [Na^+^] and [Cl^−^] vary considerably within and among individuals. Sweat [Na^+^] and [Cl^−^] values reported in the literature are typically ~ 10 to 90 mmol/L across a wide range of participants tested in various exercise and environmental conditions (Baker et al. 2009, 2018b; Barnes et al. 2019; Maughan and Shirreffs 1998; Patterson et al. 2000; Robinson and Robinson 1954; Shirreffs and Maughan 1997; Verde et al. 1982) (Table 1).

Potassium secretion occurs according to the Na^+^–K^+^–2Cl^−^ cotransport model described above. Final sweat typically has a [K^+^] similar to that of primary sweat and blood plasma (3.6–5.2 mmol/L), which may suggest that minimal changes in sweat [K^+^] occur during sweat passage along the duct (Gibinski et al. 1973). Some studies report sweat [K^+^] values with a slightly broader range (e.g., ~ 2 to 8 mmol/L) than blood plasma (Baker et al. 2009; Patterson et al. 2000; Taylor and Machado-Moreira 2013), but the underlying reasons for this discrepancy (between sweat and plasma) are unclear to date. Moreover, there have been mixed results with respect to the relation between flow rate and sweat [K^+^] (Baker 2019; Gordon and Cage 1966; Patterson et al. 2000; Sato 1977). Other papers speculate that K^+^ is secreted in the eccrine duct (Buono et al. 2016; Sato 1980), but more research is needed.

Even when testing conditions and sweat collection/analysis methodologies are standardized and well controlled, ~ 5 to 16% intra-individual day-to-day variability (CVs) in sweat [Na^+^] and [Cl^−^] is observed (Baker et al. 2009, 2018b; Hayden et al. 2004). The day-to-day CV for sweat [K^+^] is 6–19% (Baker et al. 2009). Assuming that ~ 1 to 5% of this CV is due to intra-instrument variability (Baker et al. 2014; Boulyga et al. 2007; Doorn et al. 2015; Goulet et al. 2017, 2012; Pullan et al. 2013), this suggests that the remaining up to ~ 5 to 14% CV is probably due to physiological variability.

### Other micronutrients (calcium, magnesium, iron, copper, zinc, vitamins)

Very few studies have investigated mechanisms of sweat secretion for any micronutrients other than Na^+^, Cl^−^, and K^+^. As discussed above, research concerning trace elements and vitamins in sweat is confounded by skin cell contamination. Studies that have taken measures to collect cell-poor sweat suggest that final sweat micronutrient concentrations are similar to or less than blood plasma concentrations. Sweat trace element concentrations have been reported to be much more varied than that of the blood plasma (Table 1). Because of the paucity of data, it is unknown whether this is due to varying mechanisms of secretion/reabsorption in the sweat gland or the difficulty in measuring true sweat trace element concentrations untainted by the contents of skin cells.

While Ca^2+^ ions play an important role as a second messenger in the process of sweat secretion, the mechanism of Ca^2+^ movement into the lumen of the secretory coil is not well understood. In a series of studies by Gibinski et al. (1973, 1974), radioisotopes of Ca^2+^, K^+^, and Na^+^ were administered (orally or intravenously) 4–5 h before heat exposure and their concentrations were subsequently measured in sweat. Radio-labeled K^+^ and Na^+^ appeared in sweat within minutes of administration (Gibinski et al. 1973). Radio-labeled K^+^ in sweat was similar to that of blood after ~ 1 h of sweating. By contrast, radio-labeled Ca^2+^ appearance in sweat was slow and did not exceed 5% of blood plasma concentrations (Gibinski et al. 1974). The authors suggested that the binding of Ca^2+^ may explain the difference in the dynamics of Ca^2+^ compared with K^+^ and Na^+^ appearance in sweat. Approximately 50% of plasma Ca^2+^ is bound to protein or complexed with citrate, bicarbonate, or phosphate, while the other 50% is free, ionized Ca^2+^ (Jahnen-Dechent and Ketteler 2012). Similarly, 30–45% of plasma Mg^2+^ is bound or complexed, while 55–70% is free, ionized Mg^2+^ (Jahnen-Dechent and Ketteler 2012). Charged ions such as free Ca^2+^ and Mg^2+^ are hydrophilic but small so they may be secreted readily via the paracellular route. By contrast, protein-bound fractions of Ca^2+^ and Mg^2+^ may not be as readily available for secretion and/or may occur at a slower rate. This may explain why [Ca^2+^] and [Mg^2+^] of cell-poor sweat are more similar to ionized than to total plasma [Ca^2+^] and [Mg^2+^] concentrations (Gibinski et al. 1974).

Most (~ 70%) of plasma Zn^2+^ (albumin) and nearly all (≥ ~ 95%) of plasma Cu^2+^ (ceruloplasmin) and plasma Fe^2+^ (transferrin) are bound to carrier proteins. It is unclear how these trace elements are secreted by clear cells of the eccrine gland. In other cells throughout the body divalent metal transporter 1, divalent cation transporter 1, or other carrier-mediated systems play an important role in moving Fe^2+^, Zn^2+^, and Cu^2+^ across the membrane (Anderson and Frazer 2017; Jahnen-Dechent and Ketteler 2012; McArdle 1992; Roohani et al. 2013). With regard to vitamin secretion in sweat, ascorbic acid and thiamine are large polar molecules (Table 1) and, therefore, may be secreted through a paracellular route albeit exact mechanisms are unknown. Few studies have measured water-soluble vitamin concentrations in sweat (Mickelsen and Keys 1943; Thapar et al. 1976) and in some cases interpretation is difficult due to limited methodologies (scraping) used (Tang et al. 2016). The authors are unaware of any studies reporting sweat concentrations of fat-soluble vitamins. More research is needed to understand mechanisms of trace element and vitamin movement across cell membranes of the eccrine gland secretory coil and duct.

### Metabolites

#### Glucose

There is significant interest in sweat glucose as a possible noninvasive alternative to blood glucose monitoring in diabetic patients. While glucose is present in sweat, as first demonstrated by Silvers et al. (1928), its concentration is ~ 100 × lower than that of blood glucose (Boysen et al. 1984; Jajack et al. 2018; Katchman et al. 2018; Lobitz and Mason 1948; Moyer et al. 2012; Ono et al. 2018). There is still speculation regarding the exact mechanism of glucose secretion into sweat. Most studies suggest that blood glucose is the primary source of sweat glucose (Boysen et al. 1984; Jajack et al. 2018; Moyer et al. 2012; Ono et al. 2018) albeit its large size and polarity likely limit the passage of glucose into the lumen of the eccrine gland.

Early studies on the mechanisms determining sweat glucose concentrations were often limited by analytical techniques using reducing agents that may have included sugars other than glucose. More recent studies have directly measured sweat glucose concentration. Boysen et al. (1984) collected thermally induced sweat and were the first to show a connection between blood glucose and sweat glucose concentrations. The investigators observed a concomitant rise in sweat and plasma glucose concentrations following oral and intravenous glucose administration in two participants (Boysen et al. 1984). In addition, Moyer et al. (2012) demonstrated a correlation between sweat and blood glucose in diabetic patients after pilocarpine administration. However, neither study investigated potential mechanisms of glucose secretion. Some recent papers have suggested a potential route of glucose transport that is paracellular in nature (Heikenfeld et al. 2019; Jajack et al. 2018; Katchman et al. 2018). Paracellular transport is highly regulated via tight junctions that function to limit large molecule transport (Kutchai 1998). Glucose flux in sweat has been shown to change with the modulation of tight junctions in the sweat gland epithelium (Jajack et al. 2018). Using citrate, a calcium chelator, Jajack et al. (2018) were able to modify paracellular pathways and found a > 10-fold increase in sweat glucose flux. However, it is unclear whether paracellular transport of glucose may occur in the secretory coil (Jajack et al. 2018) or duct (La Count et al. 2018) of the eccrine gland.

A recent study has suggested there may be a transcellular route by which glucose is secreted into sweat. Ono et al. (2018) measured sweat glucose concentration in patients with and without atopic dermatitis and found an increase in sweat glucose concentration in those with atopic dermatitis. The investigators also found an increased expression of glucose transporter GLUT2 mRNA, along with a potential luminal translocation in atopic dermatitis patients. However, it is unclear if the increased expression of GLUT2 or the increased sweat glucose concentration is what caused the luminal localization (Murota et al. 2018; Ono et al. 2018). Expression of sodium-dependent glucose transporters SGLT3 and SGLT4 were also observed, but it was unclear if these transporters were upregulated in atopic dermatitis patients (Ono et al. 2018). Further studies are needed to elucidate the mechanisms of glucose secretion into sweat.

#### Lactate

Plasma lactate concentration is often monitored in competitive runners/cyclists and used as a tool to design exercise training regimens and set race pace. Thus, there is some interest in sweat lactate as a more practical means to monitor exercise intensity and physiological stress in athletes. However, as discussed in more detail below, sweat lactate concentrations generally do not correlate well with that of the blood (Alvear-Ordenes et al. 2005; Fellmann et al. 1983; Green et al. 2000; Weiner and Van Heyningen 1952). This is likely due, at least in part, to lactate production by the eccrine gland itself (Astrand 1963; Gordon et al. 1971; Wolfe et al. 1970).

The presence of glycogen in eccrine glands has been shown histochemically since the early 1900s (Brunner 1906). Studies have also shown that glycogen becomes depleted with sweating and is repleted with rest (Yuyama 1935). Glycogen content and phosphatase activity in the secretory coil (Bunting et al. 1948; Shelley and Mescon 1952), combined with in vivo and in vitro studies, have led many investigators to the conclusion that lactate is produced via eccrine gland metabolism as an end-type process of glycolysis (Ament et al. 1997; Kuno 1956; Robinson and Robinson 1954; Sato 1977; Sato and Dobson 1973; Sato et al. 1989; Weiner and Van Heyningen 1952; Wolfe et al. 1970). Furthermore, a tracer study that injected ^14^C-labeled glucose and lactate indicated that the excreted lactate was derived from blood glucose rather than blood lactate (Gordon et al. 1971). Collective observations throughout the literature indicate that sweat lactate concentration typically exceeds that of blood lactate (Ament et al. 1997; Derbyshire et al. 2012; Fellmann et al. 1983; Green et al. 2000; Weiner and Van Heyningen 1952; Whitehouse 1935), especially at the onset of initial sweating when flow rates are low. There seems to be an inverse relation between flow rate and lactate concentration such that sweat lactate concentration decreases as sweating rate increases, possibly as a result of dilution (Ament et al. 1997; Astrand 1963; Buono et al. 2010; Derbyshire et al. 2012; Falk et al. 1991). Studies have found a direct relation between sweating rate and lactate excretion rate in terms of mmol/cm^2^/min (Astrand 1963; Buono et al. 2010) albeit this is not surprising given that lactate excretion rate is a function of sweating rate as well as sweat lactate concentration.

To the authors’ knowledge, no study has been performed to determine the mechanism by which lactate is transported across cell membranes of the eccrine gland and into the lumen. Gordon et al. (1971) first implied that the high rate of glycolysis in eccrine gland tissue and the production of lactate is linked to the secretory process, which seems to be corroborated by the finding that oxidative phosphorylation of plasma glucose is the main energy production route (Sato 1977; Sato and Dobson 1973). Considering that the cellular location of glycogen is likely limited to the clear cells of the secretory coil, it seems sweat lactate may be derived from the same location (Sato 1977). It has been speculated that due to a low *p*Ka and a small molecular size, lactate is capable of passive diffusion (Sato 1977; Sonner et al. 2015). However, recent transcriptomic and proteomic analysis of human eccrine cells identified similarities in gene expression between the human kidney and sweat glands, indicating that many genes expressed within the cortex of the kidney are also expressed within eccrine glands (Na et al. 2019). Of particular interest is the finding of solute carrier family (SLC) proteins previously only found in the kidneys. Lactate is actively transported by monocarboxylate transport proteins (MCT), also within the SLC gene family (Price et al. 1998), which has been linked to kidney cells located within the proximal tubule and cortex (Wang et al. 2006). It may be possible that MCT proteins are also expressed in clear cells of sweat glands and aid in the transport of lactate out of the cell. However, these mechanisms of lactate partitioning are complex and speculative and future studies are needed to better characterize lactate transport in eccrine sweat glands.

#### Ammonia

Ammonia is a waste formed mostly through bacteria in the intestines during protein digestion and used as substrate in the urea cycle. Various amino acids produced as metabolites of proteins are then converted into glutamic acid (glutamate) and subsequently deaminated by glutamate dehydrogenase in the mitochondria to produce ammonia. Ammonia is a small uncharged polar molecule typically found in higher concentrations in sweat than in blood (Table 1). The mechanisms underlying sweat ammonia concentration have been speculated for decades, beginning with Mosher (1933) and the proposal that ammonia is a byproduct of urea catabolism in eccrine glands. More recently, Czarnowski et al. (1992) have proposed instead that plasma ammonia is the main source of ammonia in sweat. In their study, ammonia and urea concentrations were measured in blood and sweat samples collected from four groups: controls, cirrhotic hyperammonemic patients, uremic patients, and a group that ingested ammonium chloride (Czarnowski et al. 1992). Increased plasma ammonia (in cirrhotic patients and from ammonium ingestion) resulted in increased sweat ammonia concentrations, but no increase in sweat urea concentrations. Furthermore, this (Czarnowski et al. 1992) and other studies (Brusilow and Gordes 1965) have found that uremic patients had increased sweat urea concentrations, but their sweat ammonia concentrations were similar to controls. Taken together, these results suggest that ammonia in sweat did not come from urea breakdown, but instead originated primarily from the plasma (Czarnowski et al. 1992).

This does not, however, explain the significantly higher ammonia concentrations typically found in sweat versus blood. Brusilow and Gordes (1968) have proposed that diffusion and ionic ammonium trapping is only the final step of ammonia secretion, as glandular production may also be a source of sweat ammonia. These authors investigated the partial pressure of ammonia (pNH_3_) in sweat samples of differing pH and suggested that alkaline sweat containing a higher pNH_3_ than blood was evidence for glandular ammonia production (Brusilow and Gordes 1968). However, isolated gland studies and direct evidence for this idea is lacking. Another hypothesis proposed by Itoh and Nakayama (1952) suggests that sweat ammonia may be derived from glutamine catabolism in the skin. The investigators isolated glutaminase I activity in human skin tissue and measured ammonia production following incubation in several amino acid solutions. In this study, only glutamine caused large increases in ammonia concentration. The large amount of amino acids found within sweat and the relatively small concentration of glutamine (Coltman et al. 1966; Dunstan et al. 2016; Gitlitz et al. 1974; Liappis et al. 1979) would seem to support this conclusion. Furthermore, several investigators have found that the skin itself has the capability to emit ammonia (Nose et al. 2005; Schmidt et al. 2013; Turner et al. 2008). In summary, while plasma ammonia plays an important role in determining sweat ammonia concentrations (Czarnowski et al. 1992; Sato 1977), the source of excess ammonia in sweat compared with blood is equivocal. Sweat ammonia concentration could be impacted by ammonia formation in skin tissue, production in the sweat gland, and/or possibly from some form of skin emission-sweat incorporation.

Given that the permeability for ammonia is similar to water (due to molecular size and polarity), Czarnowski et al. (1992) and others (Sato 1977; Sato et al. 1989) have suggested that the pH gradient between blood, sweat, and water compartments allows ammonia to passively diffuse across membranes. Due to the low pH of final sweat (~ 5 to 7), ammonia (NH_3_) molecules that diffuse into the glandular lumen protonate to ammonium (NH_4_^+^) effectively trapping the molecules from the ionization and acquired charge (Sato et al. 1989; Sonner et al. 2015). This notion is in accordance with work from Brusilow and Gordes (1968) who found a negative correlation between the sweat-to-blood ammonia concentration ratio and sweat pH. Similarly, Ament et al. (1997) found that ammonia and lactate were present in the same order of magnitude throughout exercise. Thus, lactate may be acidifying sweat, thereby establishing the pH gradient needed for ammonia flux and subsequent shift into the glandular lumen. These results suggest ammonia may pass either through or between the cells of the secretory coil by way of a passive diffusion mechanism although direct evidence from permeability studies is lacking.

#### Urea

Urea is a nitrogenous compound formed in the liver from ammonia and is the final end product of protein metabolism. Excretion of urea is accomplished primarily by the kidneys; but it is also excreted by sweat glands, most notably demonstrated by the urea frost found on the skin of uremic patients (al-Tamer et al. 1997; Sato et al. 1989). Urea is a small polar molecule and easily crosses the glandular wall and cell membrane (Komives et al. 1966; Sato et al. 1989). Thus, sweat urea is thought to be derived from the plasma (Komives et al. 1966; Sato 1977; Sato et al. 1989). However, reported sweat urea concentrations are often higher than that of the plasma in both healthy and uremic patients (Table 1 and Supplemental Table 4). Several hypotheses for the increased urea in sweat have been proposed. These include urea production (Araki and Ando 1953; Brusilow and Gordes 1968; Sato et al. 1989) or ‘insensible’ metabolism (Brusilow 1967; Rothman et al. 1949) stemming from the split of arginine to ornithine and urea via arginase activity in the eccrine gland or skin (Rothman and Sullivan 1949; Talbert et al. 1927), water resorption in the eccrine duct (Schwartz et al. 1953), and/or a finite pool of urea located in the epidermis and/or gland (Brusilow 1967; Brusilow and Gordes 1968; Gordon et al. 1976).

Urea tracer studies provide perhaps the best evidence regarding the source of urea in sweat (Brusilow 1967; Gordon et al. 1976). Brusilow (1967) found that the sweat-to-plasma ratio of ^14^C-labeled urea after oral administration was 1.08, while the ratio for total urea was 1.43. These results support the notion that urea passes readily into sweat from plasma and also suggests a non-plasma source of urea since unlabeled urea was present in final sweat. Gordon et al. (1976) reported similar results and postulated that the source of non-plasma urea was a finite pool of urea in the epidermis, presumed to be concentrated through insensible evaporation of water (Fig. 1). Further support for this hypothesis is provided by studies showing a progressive decline in the sweat urea-to-blood urea ratios to near unity during periods of profuse sweating (Komives et al. 1966). By contrast, the idea that sweat constituents become concentrated via water reabsorption in the duct lacks support (Amatruda and Welt 1953; Gordon et al. 1976; Hew-Butler et al. 2014).

While urea is a small molecule able to diffuse through and/or between cell walls, recent literature suggests that eccrine glands may express localized urea transporter subtypes (Keller et al. 2016; Na et al. 2019; Xie et al. 2017). The expression of urea transporter 1 isoform 1 (UT-1) at the transcriptome and proteome level suggests the facilitation of transmembrane urea movement (Keller et al. 2016; Xie et al. 2017). UT-A1 and UT-B1 protein expression and localization have been found in cells of both the secretory coil (clear cells) and duct (Keller et al. 2016), with significantly higher mRNA expression in uremic patients compared with normal controls (Xie et al. 2017). These findings suggest that with excess urea production, there may be an active mechanism of urea excretion through sweat. Additionally, the overlap in gene expression found between eccrine glands and kidneys may indicate that sweat is another way to excrete metabolic wastes (Na et al. 2019), albeit the effectiveness of this function remains uncertain (Baker 2019).

#### Bicarbonate

Bicarbonate is present in most body fluids and organs and plays an important role in acid–base balance. It has been proposed that bicarbonate is secreted and reabsorbed in the eccrine gland, dictating the pH of sweat in the process; although exact mechanisms have not been established (Sato 1983; Sato and Sato 1990). One proposed mechanism involves a bicarbonate/Cl^−^ exchanger (Quinton and Reddy 1989; Reddy and Quinton 1994a) in combination with a hydrogen-ATPase pump, which could create a favorable gradient for bicarbonate luminal secretion following Cl^−^ reabsorption (Patterson et al. 2000). Furthermore, a recent study has proposed a new mechanism stemming from a possible Best2 bicarbonate/Cl^−^ channel located in the dark cells of the secretory coil (Cui and Schlessinger 2015). This suggested working model may be involved in acid–base balance and sweat secretion via elevated Ca^2+^ action leading to bicarbonate secretion specifically from dark cells (Cui and Schlessinger 2015). Similarly, Cui et al. (2016) detected mRNA expression of a bicarbonate/Cl^−^ exchanger termed pendrin (SLC26a4) in isolated secretory cells of murine footpad sweat glands. This anion exchange protein may also play a role in bicarbonate secretion (Cui et al. 2016; Saint-Criq and Gray 2017).

Carbonic anhydrase, the enzyme responsible for catalyzing the reaction of carbon dioxide plus water and bicarbonate plus hydrogen, has been immunohistochemically located in both clear cells and apical lumen cells of the eccrine gland (Briggman et al. 1983; Clunes et al. 2004). It has been suggested that the appearance of both isoforms of carbonic anhydrase in clear cells indicates that the enzyme may play a role in bicarbonate secretion into the lumen (Clunes et al. 2004). Similarly, the presence of carbonic anhydrase in the apical lumen cells in combination with a luminal bicarbonate/Cl^−^ exchanger (Bonar and Casey 2008; Bovell and Quinton 2002; Quinton and Reddy 1989; Reddy and Quinton 1994a) suggests a possible route for bicarbonate reabsorption. Briefly, it has been hypothesized that the hydrogen-ATPase transports hydrogen into the lumen, creating a gradient for bicarbonate entry, which is then neutralized to carbonic acid. The carbon dioxide in combination with the carbonic anhydrase in the luminal cell cytoplasm would create a continuous cycle of bicarbonate production for Cl^−^ exchange (Quinton and Reddy 1989; Reddy and Quinton 1994a).

Bicarbonate is also thought to be reabsorbed in the sweat duct via CFTR, combined with hydrogen secretion, leading to the acidification of final sweat (Choi et al. 2001). Much like Na^+^ and Cl^−^, bicarbonate reabsorption is inversely related to sweating rate. Thus, lower sweat flow rates are associated with a lower bicarbonate concentration and lower pH of final sweat (Collins et al. 1966; Kaiser et al. 1974). While these theories are still speculative, it seems most likely that sweat pH is an artifact of a reabsorptive duct function and hydrogen ion secretion via an apical membrane hydrogen-ATPase pump activity (Bovell et al. 2000), basolateral Na^+^/H^+^ exchange (Granger et al. 2003; Kaiser et al. 1974), or bicarbonate reabsorption (Patterson et al. 2000, 2002).

#### Amino acids

Amino acids are organic compounds that combine to form proteins. The human body uses amino acids to break down food, repair body tissues, and, in some cases as fuel. In the skin, amino acids or their derivatives function as natural moisturizing factors that can act as humectants (Watabe et al. 2013). Amino acids were first identified in sweat in 1910 (Embden and Tachau 1910) and since then several more studies have isolated up to 20 different amino acids in sweat collected on the skin surface (Coltman et al. 1966; Dunstan et al. 2016; Gitlitz et al. 1974; Hier et al. 1946; Rothman and Sullivan 1949). Possible mechanisms underlying amino acid appearance in sweat may include selective secretion by the gland, selective reabsorption (Araki and Ando 1953), or a combination of both (Gitlitz et al. 1974). It may be that the transfer of amino acids from the interstitial fluid into eccrine sweat is influenced by molar volume, polarity, or ligand binding, among other factors (Gitlitz et al. 1974). More work is needed to elucidate mechanisms of sweat amino acid composition. Nonetheless, it is apparent that amino acid appearance in sweat is not merely a result of filtration from the plasma, as Hier et al. (1946) found that amino acid ingestion did not affect sweat amino acid concentrations.

The non-plasma source of amino acids in sweat is likely the skin itself, as recent research has found a strong similarity between sweat amino acid composition and natural moisturizing factors (NMF) and epidermal proteins (Mark and Harding 2013). Moreover, several NMF produced in the stratum corneum (serine, histidine, ornithine, glycine, alanine, lysine, aspartic acid) appear in higher concentrations in the sweat than blood (Dunstan et al. 2016). It may be that the stratum corneum influences the amino acid content of sweat via the hydrolysis of the epidermal protein filaggrin, present in keratohyalin granules of the acrosyringium (Langbein et al. 2005; Mark and Harding 2013). This hydrolysis would allow filaggrin-derived amino acids to diffuse into the sweat directly from the ductal lining as it passes onto the surface of the skin (Mark and Harding 2013). Another line of evidence for the hypothesis that amino acids are leached from the skin is that the amount of NMF in sweat declines as exercise duration increases (Dunstan et al. 2016). Several studies have also found an inverse relation between sweating rate and sweat amino acid concentration (Araki and Ando 1953; Dunstan et al. 2016; Gitlitz et al. 1974; Itoh and Nakayama 1952), suggesting possible dilution of amino acid concentration occurring with greater sweat (water) volume. In summary, the exact mechanism underlying the presence of amino acids in eccrine sweat is uncertain; however, it seems that skin NMF plays a large role in sweat amino acid composition. The variation in sweat amino acid profiles among studies and individuals may stem from the capacity to replenish skin NMF (Dunstan et al. 2016).

#### Ethanol

A commonly perceived function of eccrine glands is the elimination of ethanol from the body via enhanced sweating rates and increased sweat ethanol concentration. There is no experimental evidence that alcohol ingestion leads to an increase in sweating rate (Baker 2019). It does appear that sweat ethanol concentration increases linearly with ethanol ingestion, as several investigators have demonstrated a significant positive correlation between sweat and blood ethanol concentrations (Brown 1985; Buono 1999; Gamella et al. 2014; Hauke et al. 2018; Phillips and McAloon 1980). Because of its small size and hydrophilic–lipophilic nature, ethanol is easily distributed throughout body water via passive diffusion (Cederbaum 2012; Jacobsen 1952; Nyman and Palmlov 1936; Paton 2005; Pawan and Grice 1968), and is likely secreted into sweat by the same mechanism.

It is important to note, however, that while a strong relation between sweat and blood ethanol concentrations exists (Brown 1985; Gamella et al. 2014; Hauke et al. 2018), the primary mechanism of ethanol elimination is oxidation within the liver. Briefly, a combination of four alcohol dehydrogenases catalyze multiple reactions breaking ethanol into acetaldehyde, acetate, and eventually acetyl CoA (Cederbaum 2012; Paton 2005). It is through this process that 90% of ethanol is eliminated with a remaining 2–10% thought to be excreted through breath, sweat, and urine (Cederbaum 2012; Jacobsen 1952; Paton 2005).

### Others

#### Cytokines

Cytokines are small secreted proteins with pleiotropic effects important in autocrine, paracrine, and endocrine cell signaling (Zhang and An 2007). Interest in sweat cytokines likely stems from their relation to inflammation, infection, and immune response. Their large molecular weight would seem to preclude secretion into eccrine sweat; however, suggestive mechanisms have been proposed (Heikenfeld et al. 2019; Sonner et al. 2015). The presence of cytokines in human sweat was first reported by Didierjean et al. (1990). This study involved active, passive, and thermal stimulation and all samples expressed at least one form of interleukin (IL)-1α or IL-1β. Other investigators have found similar results regarding IL-1 and have also detected IL-6, IL-8, IL-10, IL-31, tumor necrosis factor (TNF)-α, and transforming growth factor (TGF)-β in sweat (Cizza et al. 2008; Dai et al. 2013; Hladek et al. 2018; Jones et al. 1995; Marques-Deak et al. 2006; Sato and Sato 1994) although IL-6 and TNF-α are not consistently detected (Dai et al. 2013; Faulkner et al. 2014).

The secretory coil, as well as straight and coiled portion of the duct, expresses immunohistochemical activity of IL-1α and IL-1β. Expression of these cytokines is found in both luminal and basal cells of the duct, but more intense staining is exhibited in luminal cells. Only the clear cells of the secretory coil express IL-1α and IL-1β (Ahmed et al. 1996; Grellner 2002; Reitamo et al. 1990; Sato and Sato 1994). Likewise, immunolabeling of the eccrine gland has shown expression of IL-6, IL-8, IL-31, TGF-β, and TNF-α (Ahmed et al. 1996; Anttila et al. 1992; Dai et al. 2013; Grellner 2002; Jones et al. 1995; Tian and Stacey 2003). It seems these cytokines follow a similar trend as IL-1, with strong staining exhibited in the clear cells of the secretory coil and in the luminal cells of the duct (Ahmed et al. 1996; Dai et al. 2013; Jones et al. 1995; Tian and Stacey 2003). Furthermore, in situ hybridization studies have found that mRNA encoding for IL-1α, IL-1β, IL-8, and TNF-α are present in the secretory coil and duct (Boehm et al. 1994; Jones et al. 1995), suggesting that these cytokines are innately expressed in eccrine glands.

While generally linked to inflammation, cytokines in sweat appear with and without associated local inflammation. Furthermore, the finding that insensible sweat cytokine concentration is correlated with plasma concentrations suggests at least a portion is derived from the circulating stores (Cizza et al. 2008; Marques-Deak et al. 2006). However, the hypothesis that cytokines are cleared from the blood via sweat is not fully supported by the current available literature. It seems more likely that these cytokines are derived from the eccrine gland itself. The intense staining pattern of clear secretory coil cells and lumen duct cells would seem to indicate a cell-associated relationship (Ahmed et al. 1996). Likewise, the elevated sweat cytokine concentrations during exercise and thermal stress (Didierjean et al. 1990; Sato and Sato 1994) combined with the regional differences in sweat cytokine concentration (Didierjean et al. 1990) would indicate stress-induced cell secretion rather than an origination from the blood. The gradual taper of IL-1 secretion in sweating lasting over 1 h might suggest the diminishing of these stores, with the presence of mRNA indicating to the ability to resynthesize following secretion (Dai et al. 2013; Murphy 1995; Sato and Sato 1994). However, given study limitations, it is not possible to rule out that cytokine sweat concentration could be the consequence of a storage function or due to the rate of synthesis by the gland.

Some have suggested that sweat cytokines play a role in pathophysiological function: hypotheses include surveillance and readiness for rapid response and cell recruitment to stress, immune, and environmental insults (Boehm et al. 1994; Didierjean et al. 1990; Tian and Stacey 2003), ductal regulatory function (Boehm et al. 1994), and further cytokine regulation in cascade-based fashion (Sato and Sato 1994). However, direct mechanisms of cytokine secretion in eccrine sweat remain speculative.

#### Cortisol

Cortisol acts as the main glucocorticoid hormone produced by the adrenal cortex and is often used to assess adrenocortical function. Release of cortisol is both spontaneous and in response to biochemical agents or psychological/physiological stimuli (Kirschbaum and Hellhammer 1989, 2000). Approximately 90% of the endogenous hormone is bound to carriers, and it is believed that the remaining 5–10% of unbound or free cortisol is biologically active on target tissues (Ekins 1990). Unbound cortisol is thought to diffuse readily into cells due to their lipid-rich cell membrane (Kirschbaum and Hellhammer 1989). This passive transport through lipid bilayer membranes is what likely allows for the detection of cortisol in many bodily fluids in addition to blood, such as saliva (Kirschbaum and Hellhammer 1989; Perogamvros et al. 2010; Umeda et al. 1981), sweat (Jenkins et al. 1969; Jia et al. 2016; Lewis and Thorn 1955; Nichols and Miller 1948; Russell et al. 2014), and human hair (Raul et al. 2004).

The most compelling study on sweat cortisol mechanisms was published by Jenkins et al. (1969), who used an intravenous tracer method to track the movement of cortisol from blood to sweat. In this study, participants received an intravenous infusion of ^14^C labeled-cortisol prior to thermally induced sweating. Only 0.024–0.073% of the labeled cortisol appeared in sweat as the unchanged compound or unconjugated metabolites. Based on radioactivity of the samples, a larger quantity of unbound cortisol was secreted in sweat compared with cortisol tightly bound to plasma proteins. Unconjugated cortisone concentrations in sweat were similar to that of plasma but less than plasma cortisol (Jenkins et al. 1969). These results are in agreement with the findings of Lewis and Thorn (1955), who reported that low concentrations (< 8 μg per 100 ml) of cortisol and cortisone were present in sweat. Interestingly, a low concentration of cortisol in sweat remained even following a 25 mg injection of exogenous cortisol prior to exercise, while urine cortisol excretion increased approximately threefold (Lewis and Thorn 1955).

Jenkins et al. (1969) speculated that, because of the comparable histology of eccrine glands and parotid glands, a similar enzymatic conversion of cortisol to cortisone may take place during the thermal sweat secretion process. These results suggest that protein-bound fractions may not be readily available for secretion and or conversion, whereas the smaller unbound or unconjugated fractions are able to diffuse into secretory fluid. While this concept has been investigated in saliva, it is largely speculative in eccrine glands. However, some evidence, stemming from in vitro metabolic studies of human skin, seems to suggest a transformation of cortisol may take place (Hsia et al. 1964). Furthermore, an enzyme recognized to convert cortisol to cortisone (11β-hydroxysteroid-dehydrogenase Type 2) has been immunolocalized to luminal duct cells of eccrine glands (Hirasawa et al. 1997; Kenouch et al. 1994; Smith et al. 1996), but found to be lacking in hair follicles, epidermis and sebaceous glands (Hirasawa et al. 1997; Smith et al. 1996). The localization of 11β-hydroxysteroid-dehydrogenase Type 2 to eccrine glands and the finding of cortisone and cortisol in hair suggest an incorporation following active or passive diffusion from eccrine sweat (Raul et al. 2004). Incorporation of molecules into human hair depends on basicity, melanin affinity, and lipophilicity (Raul et al. 2004) resulting in three potential mechanisms for incorporation: active or passive diffusion from blood into the growing follicle, diffusion from secretions during hair shaft formation, and external sources following shaft formation (Kintz et al. 2000; Wennig 2000). If diffusion is responsible for cortisol incorporation into hair, it seems possible that a similar process may explain cortisol diffusion into eccrine sweat. Nonetheless, this rationale is still speculative and more research is needed to understand the mechanisms of cortisol movement.

## Differences in sweat composition with passive versus active sweating

There are two main ways by which sweat secretion is stimulated: sweating induced by passive and active methods. Passive sweating occurs while the participant is at rest and can be induced by pharmacological manipulation or by increasing the temperature of the surrounding air (e.g., dry heat or sauna bathing). Active sweating refers to exercise-induced sweat secretion. Pharmacologically induced sweating involves the use of a small electrical current (iontophoresis) to propel charged cholinergic agonists (e.g., pilocarpine or methacholine) transdermally to stimulate the muscarinic receptors on the sweat glands (Gibson and Cooke 1959; Webster 1983). Pilocarpine iontophoresis is commonly used in research and is the gold-standard sweat-testing method for cystic fibrosis (Mishra et al. 2005). It is often the method of choice from a practical perspective since it is relatively quick and circumvents the discomfort of heat stress and/or physical exercise. However, it is important to note that pharmacological methods induce sweat gland secretion only via local cholinergic stimulation. With exercise and/or passive heat stress, other local (e.g., skin temperature, skin blood flow, adrenergic stimulation, and other neuromodulators) and central mediators (e.g., body core temperature, central command, and exercise pressor reflex) are involved in stimulation of sweating (Shibasaki and Crandall 2010).

Several studies have compared the local sudomotor response to pharmacological stimulation, passive heat stress, and/or exercise. Forearm sweating rate has been reported to be higher with exercise and/or thermal stress compared with pilocarpine iontophoresis (Hjortskov et al. 1995; Vimieiro-Gomes et al. 2005). The reason for the discrepancy between methods could be related to the different mechanisms involved in sweat stimulation (Vimieiro-Gomes et al. 2005). In general, higher sweating rates have been reported with exercise than passive heating (Taylor and Machado-Moreira 2013) albeit the differences may be attenuated when heat production, body core temperature, and skin temperatures are consistent between methods (Nielsen and Nielsen 1965).

### Sodium chloride and other micronutrients

Supplemental Table 3 summarizes the literature comparing the effect of different sweat stimulation methodologies on sweat composition. The results have been mixed and difficult to interpret, due in part to a lack of statistical analysis in some of the early papers. Separate studies have found higher (Ikai et al. 1969), lower (Fukumoto et al. 1988; Kozlowski and Saltin 1964), or similar (Verde et al. 1982) sweat [Na^+^] and [Cl^−^] with exercise versus passive heat stress. Likewise, sweat [Na^+^] and [Cl^−^] have been reported to be higher (Collins 1962; Sato et al. 1970; Shwachman and Antonowicz 1962) or similar (di Sant'Agnese and Powell 1962; Shwachman and Antonowicz 1962) with pharmacological stimulation versus passive heating. As shown in Supplemental Table 3, results were also equivocal for sweat [K^+^], [Ca^2+^], and [Mg^2+^] and no data to the authors’ knowledge are available for Zn^2+^, Cu^2+^, or vitamins. In most of these studies, sweating rate was not standardized or not reported. Even when sweating rate was matched between exercise and passive heating the impact on sweat [Na^+^] and [Cl^−^] was inconsistent (Kozlowski and Saltin 1964; Verde et al. 1982). Some studies collected sweat from different body regions (di Sant'Agnese and Powell 1962) or via different techniques (Shwachman and Antonowicz 1962) between stimulation methods, which further confound the interpretation of results.

Passive and active mechanisms of sweat stimulation have also been compared in studies measuring maximum ion reabsorption rates. Recently, Gerrett et al. (2018) compared the effects of exercise and passive heating on sweat gland ion reabsorption rates on the forearm, chest, and lower back by determining the sweating rate threshold for increasing galvanic skin conductance (Amano et al. 2016). While sweat ion concentrations were not measured, this study found that maximum ion reabsorption rates were higher during moderate-intensity exercise (60% *V*O_2max_ for 30 min) than passive heating (immersion of lower legs in 43 °C water bath for 30 min). These results suggest that factors associated with physical activity may influence the rate of Na^+^ and Cl^−^ reabsorption in the eccrine duct (Gerrett et al. 2018). More work is needed to clarify whether exercise mediates enhanced ion reabsorption rates via thermal (body core temperature or mean skin temperature) and/or nonthermal (sympathetic or hormonal) mechanisms.

Discrepancies between methods of sweat stimulation can confound interpretation of studies investigating the impact of various host or external factors on sweat electrolyte concentrations. For example, one study found lower local sweating rates and a higher sweat [Na^+^] on tattooed skin than contralateral non-tattooed skin when stimulated by pilocarpine iontophoresis (Luetkemeier et al. 2017). However, in another study, skin tattoos did not alter local sweating rate or sweat [Na^+^] of exercise-induced sweating (Rogers et al. 2019). As expected, sweating rates were much larger in the exercise study (mean of 1.19 mg/cm^2^/min) than the pilocarpine iontophoresis study (means of 0.18 and 0.35 mg/cm^2^/min), which reiterates the mechanistic and practical differences between active and passive pharmacological sweat stimulation.

Taken together, the ecological validity of sweat solute concentrations of pharmacological sweat is questionable. As such, stimulation methodology should be carefully considered when designing research and interpreting sweat composition results. The methods used to induce sweating should fit the purpose of sweat testing and intended application of the data. For example, if the intent is to use the results to inform athletes on their individual sweat electrolyte losses, then the sweat testing should be conducted during exercise and in conditions (i.e., thermal environment) specific/relevant to their sport (Baker 2017).

### Metabolites and cytokines

As shown in Supplemental Table 3, most of the literature suggests that sweat metabolite concentrations are higher with active versus passive sweating. For instance, concentrations of lactate (Astrand 1963; Mitsubayashi et al. 1994), ammonia (Mitsubayashi et al. 1994), amino acids (Liappis and Hungerland 1972), creatinine and urea nitrogen (Fukumoto et al. 1988) have been higher in sweat when stimulated via exercise as compared with passive heating. Most amino acid (Souza et al. 2018), alcohol, carbohydrate, and fatty acid (Delgado-Povedano et al. 2018) concentrations have been higher with exercise than pharmacologically induced sweating. However, there are exceptions, as one study found higher concentrations of lipid mediators in pharmacological sweat than exercise-induced sweat (Agrawal et al. 2018) and another study reported no differences in lactate concentration between exercise and passive heating (Fellmann et al. 1983). Sweat pH does not seem to vary between active and passive sweating, as long as the local environment (ventilation and humidity) is standardized between stimulation methods (Talbert 1919, 1922). Few studies have compared the two types of passive sweating, but available data suggest that lactate (Collins 1962) and amino acid (Souza et al. 2018) concentrations may be higher with passive heating than pharmacological sweat.

With respect to underlying mechanisms, it is interesting to note that the higher sweat lactate concentrations were generally associated with lower sweating rates (Astrand 1963; Collins 1962). This is consistent with the notion that sweat lactate concentrations become diluted with higher volumes of sweat (Buono et al. 2010). However, many of the other studies did not standardize sweating rate and/or anatomical location of sweat collection between methods. It is, therefore, difficult to determine the reason for higher concentrations of other metabolites with active versus passive sweating.

Only one study has compared the effects of different stimulation techniques on sweat cytokine concentrations. Didierjean et al. (1990) measured IL-1α and IL-1β in sweat collected from the hand after stimulation via passive heating, sauna bathing, or spontaneous sweating. Both IL-1 cytokines were significantly higher with passive heating (Supplemental Table 3), which was also associated with maximum sweat induction (Didierjean et al. 1990). Because epidermal contamination was avoided (via use of the anaerobic technique with a petroleum barrier), the authors concluded that at least some of the IL-1 may have originated from the sweat glands themselves (Didierjean et al. 1990). However, it is unclear whether the differences in sweat IL-1 concentrations were due to the method of stimulation or factors (e.g., increased gland stress) associated with profuse sweating.

## Effects of heat acclimation, diet, and physical training on sweat composition

### Sodium chloride and other micronutrients

It is well established that heat acclimation results in a significant enhancement in the rate of sweat secretion by eccrine glands, resulting in improved tolerance to passive and active heat stress (Allan and Wilson 1971; Kirby and Convertino 1986; Pandolf et al. 1988). Partial acclimation occurs with passive heat stress or exercise training, but exposure to both exercise and heat stress is required to achieve full heat acclimation (Saat et al. 2005; Tipton et al. 2008). In addition, heat acclimation is usually associated with a decrease in sweat [Na^+^] and [Cl^−^]. The decrease in sweat [Na^+^] and [Cl^−^] begins after 2–3 consecutive days of heat exposure and continues over time (Buono et al. 2018; Karlsen et al. 2015), resulting in an up to 30–60% decrease after 7–10 days (Allan and Wilson 1971; Buono et al. 2007, 2018; Chinevere et al. 2008; Johnson et al. 1944; Karlsen et al. 2015; Kirby and Convertino 1986; Nielsen et al. 1997; Robinson et al. 1950). Seasonal variation in sweat [Na^+^] has also been reported to be ~ 30 to 60% decrease from winter to summer (Bates and Miller 2008; Inoue et al. 1995). The linear relation between sweat flow rate and sweat [Na^+^] (discussed above) is maintained with heat acclimation. However, there is a downward shift in the regression line such that at any given sweating rate on the forearm, heat acclimation results in significantly lower forearm sweat [Na^+^] (Buono et al. 2007). The decrease in sweat [Na^+^] and [Cl^−^] despite an increase in sweating rate can be explained by the contrasting effects of acute changes in sweat flow rate versus the longer term adaptations in the sweat gland that occur with heat acclimation. The physiological mechanism underlying the decreased sweat [Na^+^] and [Cl^−^] with heat acclimation is related to alterations in the hormonal control of Na^+^ reabsorption by aldosterone, possibly increased sensitivity of the eccrine gland to circulating aldosterone concentrations (Kirby and Convertino 1986). As described above, aldosterone influences Na^+^ reabsorption by increasing the activity of Na^+^–K^+^-ATPase on the basolateral membrane in the eccrine sweat duct (Ladell and Shephard 1961; Sato and Dobson 1973).

It is important to note that a salt deficit is required to stimulate enhancement in NaCl reabsorption. Studies have found no change or a marginal increase in sweat [Na^+^] and [Cl^−^] when salt intake is sufficient to replace sweat electrolyte losses incurred during the heat acclimation protocol, (Armstrong et al. 1985a; Eichner 2008; McCance 1938; Weiner and Van Heyningen 1952). This finding is in line with the notion that NaCl conservation by the sweat glands is mediated by circulating aldosterone. Moreover, Yoshida et al. (2006) found that individual variations in sweat [Na^+^] during exercise were correlated with resting plasma aldosterone concentration, but not to plasma aldosterone during exercise. Therefore, the genomic action of aldosterone (influenced by chronic NaCl balance as a result of heat acclimation and diet) may have a stronger impact on inter-individual variations in sweat [Na^+^] than the rapid non-genomic action of aldosterone (influenced by acute exercise and dehydration) (Yoshida et al. 2006). This concept is supported by two recent studies that induced plasma aldosterone changes via dietary Na^+^ restriction for 3–5 days (Braconnier et al. 2019; McCubbin et al. 2019). McCubbin et al. (2019) reported a significant negative correlation between resting pre-exercise plasma aldosterone and sweat [Na^+^] measured during a subsequent bout of 2-h exercise in endurance athletes. Braconnier et al. (2019) also found that [Na^+^] of passive sweat (stimulated via pilocarpine iontophoresis) was negatively correlated with plasma aldosterone concentration in healthy normotensive participants.

The changes in sweat [Na^+^] and [Cl^−^] with altered dietary salt intake have been extensively reviewed in recent publications (Baker 2019; McCubbin and Costa 2018) and, therefore, will not be discussed in detail here. In brief, most (Allsopp et al. 1998; Armstrong et al. 1985a; Braconnier et al. 2019; Costa et al. 1969; Hargreaves et al. 1989; Komives et al. 1966; McCance 1938; McCubbin et al. 2019; Sigal and Dobson 1968; Weiner and Van Heyningen 1952) but not all (Costill et al. 1975; Koenders et al. 2017; Konikoff et al. 1986; Robinson et al. 1956) studies have shown that dietary Na^+^ restriction is associated with a decrease in sweat [Na^+^] and [Cl^−^]. The mixed results may be explained in part by methodological differences among studies, including the duration and degree of dietary manipulation. Changes in sweat [Na^+^] and [Cl^−^] seem to be less likely when salt consumption is altered by smaller (and perhaps more realistic) amounts (Costill et al. 1975; Koenders et al. 2017) or for a short period of time (less than 3 days, including just before/during exercise) (Hamouti et al. 2012; Koenders et al. 2017; Konikoff et al. 1986; Robinson et al. 1956, 1955). This result is perhaps not surprising based on the time course of sweat gland responsiveness to changes in aldosterone and the notion that genomic effects of aldosterone on sweat [Na^+^] are stronger than nongenomic actions.

Relatively few studies have tested the effect of physical training on sweat composition. This may be due in part to the difficulty in separating the effects of training from heat acclimation, since regular physical exercise elicits partial heat acclimation. The available studies suggest that aerobic training is associated with an increased sweat flow rate related to an increased cholinergic sensitivity and decreased threshold for sweat onset (Araki et al. 1981; Buono et al. 1991; Buono and Sjoholm 1988; Greenleaf et al. 1972; Inoue et al. 1999). However, the effect on sweat [Na^+^] and [Cl^−^] is less clear because no longitudinal studies are available. To the authors’ knowledge, mostly cross-sectional studies comparing groups with different aerobic capacities have been conducted to date. For example, Araki et al. (1981) measured sweat [Cl^−^] from the upper back of trained and untrained women during 2 h cycling at fixed absolute workloads (79 and 160 Watts) in a hot, humid, still-air environment. Sweat [Cl^−^] was significantly lower in trained participants at both work rates in both winter and summer test sessions. However, the authors also reported hidromeiosis in the trained group, which could explain in part their lower sweat [Cl^−^] (Araki et al. 1981). In another study, Henkin et al. (2010) measured sweat [Na^+^] and [Cl^−^] from the scapula of swimmers (*V*O_2max_  = 54.2 ± 5.7 ml/kg/min), runners (60.5 ± 5.8 ml/kg/min), and non-athletes (45.2 ± 2.9 ml/kg/min) during 30 min cycling in the heat at a fixed relative intensity of 65–75% maximal heart rate. Despite the significantly higher aerobic capacity and 50% higher sweating rate of the swimmers compared with the non-athletes there were no differences in sweat [Na^+^] or [Cl^−^]. By contrast, sweat [Na^+^] and [Cl^−^] were significantly lower in the runners than swimmers and non-athletes (Henkin et al. 2010). This may suggest potential NaCl conservation by the sweat glands with training, but the confounding effect of partial heat acclimation cannot be ruled out in this study since it was conducted in Brazil in the later winter where outdoor temperature reached 24 °C (Henkin et al. 2010).

Hamouti et al. (2011) measured sweat [Na^+^] from the lower back of trained (*V*O_2peak_ = 4.0 ± 0.8 L/min) and untrained (*V*O_2peak_ = 3.4 ± 0.7 L/min) participants during three bouts (40, 60, and 80% *V*O_2peak_) of cycling in the heat. As expected, sweat [Na^+^] increased with an increase in workload for both trained and untrained participants. Sweat [Na^+^] tended to be higher in trained versus untrained participants at the two higher workloads. However, local sweating rate was also higher in the trained participants (since absolute workloads were higher in trained vs. untrained). When sweat [Na^+^] was normalized for local sweating rate (which accounts for lack of standardizing absolute workload), there were no differences between groups (Hamouti et al. 2011), thus suggesting that higher sweat [Na^+^] in trained individuals was a function of higher sweat flow rate rather than higher sweat [Na^+^] per se. Taken together these cross-sectional studies suggest that elevated aerobic fitness is not associated with enhanced Na^+^ reabsorption in the sweat gland. Similar conclusions have been drawn from a sweat gland training study involving 2 h of sweating induced by twice daily intradermal injection of acetyl-β-methylcholine for 10–18 days (Johnson et al. 1969).

Recently, Amano et al. (2017) measured maximum sweat ion reabsorption rates (Amano et al. 2016) during exercise in distance runners (*V*O_2max_ = 59.1 ± 1.4 ml/kg/min), sprinters (*V*O_2max_ = 43.3 ± 1.2 ml/kg/min), and untrained (*V*O_2max_ = 38.0 ± 2.2 ml/kg/min) participants. They found enhanced maximum sweat ion reabsorption rates on the back region of distance runners and sprinters compared with untrained individuals, but no differences between distance runners and sprinters (Amano et al. 2017). Furthermore, there were no differences in sweat ion reabsorption rates among groups on the forearm and actual sweat ion concentrations were not reported (Amano et al. 2017). Considering the mixed results among studies and paucity of longitudinal data, more research is needed to clarify the effects of physical training on sweat composition. Future research should assess sweat electrolyte concentrations with repeated physical training in a longitudinal study design, as parallel design studies comparing well-trained to moderately- or poorly trained participants cannot control for potential confounding factors that may impact sweat composition.

Another question of interest is how do sweat electrolyte concentrations change throughout the course of a single bout of physical activity? Prolonged heavy sweating and humid environments associated with elevated skin wettedness can lead to hidromeiosis, a condition that causes a gradual decline in sweating rate (Collins and Weiner 1962). The mechanism of suppressed sweat flow rate with hidromeiosis is thought to be due to mechanical occlusion of sweat ducts as a result of swelling of the keratinized layer around the duct (Brown and Sargent 1965). It is unclear whether sweat suppression impacts sweat [Na^+^], [Cl^−^], or [K^+^], as most hidromeiosis studies have not included sweat electrolyte measurements. Interestingly, with heat acclimation the sweat glands become resistant to hidromeiosis such that higher sweating rates can be maintained (Ogawa et al. 1982; Taylor 2014). It is also important to note that studies have reported no decline in sweating rate and no change in sweat [Na^+^] or [K^+^] throughout 3–7 h of exercise of low intensity (e.g., walking) and light sweating (0.3–0.6 L/h) (Ely et al. 2011; Montain et al. 2007).

The effects of heat acclimation, physical training, and diet on sweat trace mineral concentrations have been studied to a lesser extent than Na^+^ and Cl^−^. Some initial studies seemed to indicate a conservation of sweat trace mineral concentrations with heat acclimation (Chinevere et al. 2008; Hoshi et al. 2002; Klesges et al. 1996) and even throughout the course of a single bout of exercise (DeRuisseau et al. 2002; Montain et al. 2007; Waller and Haymes 1996). However, Ely et al. (2013) determined that the decline in sweat mineral concentrations in previous studies was likely an artifact of epidermal contamination when using the arm bag technique and/or not pre-washing/cleaning the skin at the site of collection (Ely et al. 2011). It may be that progressive flushing of mineral residue lying on the skin surface with repeated profuse sweating may have contributed to the decrease in sweat mineral concentrations in previous studies (Ely et al. 2013, 2011). There is little if any information available to suggest that physical training impacts sweat trace mineral or vitamin concentrations. Several studies have investigated the impact of acute supplementation or chronic dietary intake of trace minerals and vitamins on sweat composition. Most studies, particularly those in healthy individuals with no known mineral or vitamin deficiencies, have reported no association between dietary intake and sweat concentrations for Zn^2+^, Fe^2+^, Ca^2+^, Cu^2+^ or ascorbic acid (DeRuisseau et al. 2002; Jacob et al. 1981; Lugg and Ellis 1954; Mitchell and Hamilton 1949; Vellar 1968b; Wheeler et al. 1973).

### Metabolites

The effects of heat acclimation or physical training on cytokine, immunoglobulin, or cortisol in human sweat have not been researched. A few studies have investigated sweat lactate, amino acid, and urea albeit with equivocal results regarding the impact of heat acclimation and physical training on these metabolites. Some (Fellmann et al. 1983; Lamont 1987; Pilardeau et al. 1988), but not all (Green et al. 2004) studies suggest that a higher fitness level is associated with lower sweat lactate concentrations. However, these results may be confounded by differences in sweating rate between groups. Lower sweat gland metabolism with lower sweat flow rates would result in less lactate production (Pilardeau et al. 1988). On the other hand, there could be a dilution effect of increased sweating rates (with physical training and/or increased absolute exercise intensity) on sweat lactate concentrations (Lamont 1987). Similarly, Liappis et al. (1979) found that total amino acid concentrations in sweat were significantly lower in trained (physically active for an average of 9 h per week) than untrained (average of 1 h of sports per week) men during 15 min of cycling at 150 W while covered with a plastic blanket (12,797 vs. 24,855 µmol/L, respectively). The authors speculated that regular stimulation of sweating via physical training caused an adaptation in the sweat glands to limit excretion of essential amino acids (Liappis et al. 1979). However, sweating rate was not standardized between groups, as sample volume collected from the forearms ranged from 1 to 6 mL in 15 min (Liappis et al. 1979). Therefore, it is possible that the more fit individuals had a higher sweat volume that could have diluted the amino acid concentrations.

Limited data on heat acclimation and sweat metabolite concentrations are available. Weiner and Heyningen (1949) found that lactate concentrations in arm bag sweat decreased with 20 -day acclimation in one participant. However, in a follow-up study, the investigators found no changes in whole body lactate concentration with heat acclimation, despite an increase in sweating rate (Weiner and Van Heyningen 1952). The literature on heat acclimation and sweat urea has reported mixed results between studies (Robinson and Robinson 1954) and significant inter-individual variability (McCance 1938). To date, no study has investigated the effects of heat acclimation or physical fitness on sweat ammonia concentrations. Physical strain and muscular activity are known to increase blood ammonia due to metabolic degradation and purine nucleotide cycle activity (Mitsubayashi et al. 1994; Schulz and Heck 2003). Endurance-trained individuals produce less ammonia during submaximal exercise (Holloszy and Coyle 1984). In addition, since ammonia is thought to passively diffuse from the plasma into sweat (Sato et al. 1989), it may be logical to hypothesize that sweat ammonia concentrations should be lower in trained versus untrained participants. Still, this idea is speculative, and confounded by the impact of sweating rate and possible ammonia contamination from apocrine and sebaceous glands. More work is needed to understand how sweat ammonia, lactate, and other metabolites are impacted by changes in physical training and heat acclimation if they are to be used as a biomarker for fitness, training, or performance purposes.

## Correlations between constituent concentrations in sweat versus blood

### Sodium chloride and other micronutrients

Supplemental Table 4 shows a summary of published studies comparing constituent concentrations in sweat versus blood. Although there is a long list of studies, few of them were well-controlled, adequately powered, and reported correlation and statistical results. Three of six studies suggested there was a significant positive correlation between sweat and blood [Na^+^] or [Cl^−^], but only for a portion of the data set (Hew-Butler et al. 2010; Robinson et al. 1956; Talbert and Haugen 1927). The other three studies found no correlation between sweat and blood [Na^+^] or [Cl^−^] (Johnson et al. 1944; McCubbin et al. 2019; Mickelsen and Keys 1943). Thus, based on the available literature to date, there is no clear evidence of a correlation between sweat and blood for [Na^+^] and [Cl^−^]. This is perhaps not surprising because, even though primary sweat is isotonic with the blood, the rate of reabsorption of Na^+^ and Cl^−^ in the eccrine duct is independent of plasma electrolyte concentrations. For example, acute changes in sweating rate alone can result in up to threefold changes in final sweat [Na^+^] (Buono et al. 2008). Additionally, in a well-controlled study in 15 male endurance athletes, McCubbin et al. (2019) found a significant correlation between pre-exercise plasma aldosterone and sweat [Na^+^] but no correlation between plasma [Na^+^] and sweat [Na^+^] (McCubbin et al. 2019). This and other studies (discussed above) suggest that factors impacting resting plasma aldosterone play an important role in determining final sweat [Na^+^] (Braconnier et al. 2019; Yoshida et al. 2006).

Few studies have compared concentrations of other micronutrients in sweat and blood; nonetheless, most have reported no correlations for Fe (Paulev et al. 1983; Vellar 1968a) or ascorbic acid (Mickelsen and Keys 1943). This may be due in part to the methodological issues, such as inconsistency in accounting for skin contamination. Interestingly, the only significant correlation reported was between cell-free sweat [Fe^2+^] and serum [Fe^2+^] albeit the correlation coefficient was small (*r* = 0.37) (Vellar 1968a). These results taken together with the lack of association between dietary micronutrient intake and corresponding sweat micronutrient concentrations suggest little support for using sweat as a surrogate for blood.

Some have suggested that sweat electrolyte concentrations can be used as a biomarker to detect dehydration or predict sweating rate (Alizadeh et al. 2018; Gao et al. 2016; Rose et al. 2015; Zhou et al. 2016); however, studies measuring sweat electrolyte concentrations alongside changes in hydration status have found mixed results. Sweat [Na^+^], [Cl^−^], and [K^+^] have been shown to increase (Lichton 1957; Morgan et al. 2004), decrease (Armstrong et al. 1985b; Costill et al. 1976), or not change (Amatruda and Welt 1953; Morgan et al. 2004; Robinson et al. 1956; Walsh et al. 1994) in response to dehydration during exercise and/or heat stress. Furthermore, studies have demonstrated significant changes in sweat [Na^+^], [Cl^−^], and/or [K^+^] in response to variations in exercise intensity (Baker et al. 2019), environment (Dziedzic et al. 2014), or diet (McCubbin et al. 2019) despite no significant differences in hydration status during exercise. Moreover, as discussed above, the correlation between sweating rate and sweat [Na^+^] for between-participant comparisons is poor (Baker et al. 2018b; Patterson et al. 2000).

### Metabolites and cytokines

The correlation between blood and sweat metabolite concentrations are of recent interest due to novel biotechnological advances enabling local metabolite detection from micro-sampling devices (Hauke et al. 2018; Heikenfeld et al. 2018). Supplemental Table 4 summarizes studies that have tested the utility of sweat as a biomarker for changes in blood metabolite and cytokine concentrations. The results for urea, ammonia, bicarbonate, amino acids, and glucose have been mixed at best. Moyer et al. (2012) found a significant correlation between sweat and blood glucose concentration in diabetic patients in whom the range in blood glucose was quite broad (~ 60 to 360 mg/dl). Another study found a significant correlation in diabetic patients, but not healthy participants (Nyein et al. 2019). These results suggest that sweat glucose may be sensitive to large changes in blood glucose, but perhaps not to relatively small changes (e.g., in non-diabetics). In fact, there is currently little evidence of a correlation between sweat and blood for glucose or other metabolites such as lactate, amino acids, and urea in healthy individuals at rest or during exercise (Supplemental Table 4). This is due in part to significant methodological issues, such as sub-optimal analytical technique (Silvers et al. 1928), very low number of participants, or unclear/incomplete analysis and reporting of results (see Supplemental Table 4). One study (Alvear-Ordenes et al. 2005) did find a significant correlation between sweat and blood in rugby players, but metabolite concentrations in sweat only accounted for 7% (ammonia) or 45% (urea) of the variation in blood concentrations. More well-controlled correlation studies are needed.

Several studies listed in Supplemental Table 4 did not report correlation results but observed the change in sweat metabolite concentration after manipulation of blood concentrations via substrate ingestion (glucose, amino acids, ammonium chloride, urea, or bicarbonate) or having participants perform exercise of various intensities (lactate, ammonia). In the ingestion studies, an increase in blood metabolite concentrations was sometimes (Boysen et al. 1984; Czarnowski et al. 1992; Komives et al. 1966) but not always (Ament et al. 1997; Hier et al. 1946; Patterson et al. 2002) associated with a significant concomitant rise in sweat concentrations. In one study, significant increases in sweat glucose concentration were observed in two participants in response to infusion and ingestion of a glucose bolus, which caused an increase in blood glucose from 60 to 360 mg/dl (Boysen et al. 1984). Again, it is still unclear if smaller changes in blood glucose would elicit measurable concomitant changes in sweat glucose. An increase in blood lactate and ammonia concentrations during exercise has been associated with a decrease (Ament et al. 1997) or no change (Alvear-Ordenes et al. 2005; Green et al. 2000; Weiner and Van Heyningen 1952) in corresponding sweat lactate and ammonia concentrations. These and other studies (Buono et al. 2010) illustrate the lack of support for using sweat lactate or ammonia as biomarker for exercise intensity.

On the other hand, a few studies have consistently reported a strong correlation (Gamella et al. 2014; Hauke et al. 2018; Phillips and McAloon 1980) and close to 1:1 ratio (Buono 1999) for ethanol concentrations in sweat compared with blood. This may be explained by the straightforward mechanism of ethanol secretion (passive diffusion, as discussed above) and the low likelihood of surface contamination or eccrine gland production. Therefore, sweat ethanol could possibly be used as surrogate for blood ethanol concentration albeit breathalyzers are already available as a non-invasive practical alternative to blood. Finally, there is some interest in sweat cytokines and cortisol as biomarkers for health and wellness (inflammation, stress, etc.). Two studies by the same group (Cizza et al. 2008; Marques-Deak et al. 2006) have found significant correlations between 24-h insensible sweat and plasma cytokine concentrations. More work is needed, however, to corroborate these findings and determine the utility of sweat cytokines for monitoring healthy individuals or patient populations. No studies have compared cortisol concentrations in sweat and blood.

## Conclusions

It is clear that final sweat composition is not only influenced by extracellular solute concentrations, but also mechanisms of secretion and/or reabsorption, sweat flow rate, byproducts of sweat gland metabolism, skin surface contamination from desquamated epidermal cells, and sebum secretions, among other factors. Thus, the concentrations of ions, metabolites, and other constituents in sweat are often much different from that of the blood. Some solutes are relatively dilute in sweat because of reabsorption in the duct (Na^+^, Cl^−^, bicarbonate), others because of limitations in transport across/between cells of the eccrine gland (glucose, cytokines, cortisol). When solutes appear in final sweat at higher concentrations than that of blood, the contamination could be derived from sweat glands (lactate), skin cells (trace minerals, amino acids, cortisol), or both (urea, ammonia, cytokines). Furthermore, significant correlations between sweat and blood have not been established for most constituents, with the exception of ethanol. With respect to sweat diagnostics, it is well accepted that high sweat Na^+^ and Cl^−^ concentrations are useful as a screening tool for cystic fibrosis. However, sweat electrolyte concentrations are not predictive of hydration status or sweating rate. In addition, sweat metabolite concentrations are not a reliable biomarker for exercise intensity or other physiological stressors. To date, glucose, cytokine, and cortisol research is too limited to suggest that sweat is a useful proxy for blood.

More research is needed; therefore, the next section outlines suggestions for future investigations. Addressing these fundamental questions and methodological issues will help elucidate the potential utility of sweat as a surrogate for blood and/or as a biomarker for physiological/nutritional status.

## Suggestions for future research

Future research concerning eccrine sweat composition should carefully consider the following methodological suggestions:

- Use sweat stimulation methods that are fit for the intended research question and application of the data (e.g., local vs. whole body, pharmacological vs. thermal, passive heat vs. exercise).
- Thoroughly clean the skin and allow ≥ 20 min of sweating prior to sample collection.
- Avoid collection methods prone to skin surface contamination (arm bag technique, scraping or dripping methods).
- Avoid collecting sweat from regions high in sebaceous and apocrine glands.
- Use an oil barrier to avoid skin surface contamination and/or centrifuge the sweat sample and separate the supernatant to isolate cell-free sweat.
- Standardize, measure, and report corresponding sweating rate data. Consider reporting total sweat constituent flux (concentration × sweating rate).
- Use filter paper or the sweat pouch method for instantaneous collection of small volumes of sweat when the protocol calls for serial measurements or synchronization of blood and sweat samples.

The following research questions represent gaps in the literature:

- Normative data and body mapping (local and whole body) for sweat mineral, metabolite, cytokine, and cortisol concentrations.
- Effect of heat acclimatization and physical training status on sweat composition.
- Correlation between sweat and blood constituent concentrations using well-controlled, adequately powered studies that report statistical analysis results and scatterplots of individual data.
- Mechanisms of secretion in the eccrine gland, especially for constituents where studies are limited or equivocal to date, such as glucose, ammonia, bicarbonate, cytokines, and cortisol, among others.
- Immunohistochemical, transcriptomic, and proteomic identification of active transport channels or transporters within isolated eccrine sweat glands.
- Potential links between the mechanisms of sweating and the physiology of other secretory/excretory processes (e.g., renal system, tear fluid, and saliva).

## Electronic supplementary material

Below is the link to the electronic supplementary material.

Supplementary file1 (DOCX 15 kb)

Supplementary file2 (DOCX 21 kb)

Supplementary file3 (DOCX 27 kb)

Supplementary file4 (DOCX 29 kb)

## Abbreviations

ACh: Acetylcholine
ATP: Adenosine triphosphate
AQP: Aquaporin
Best: Bestrophin
BK: Large conductance Ca^2^^+^-activated K^+^ channel
Ca^2^^+^: Calcium
CFTR: Cystic fibrosis transmembrane conductance regulator
Cl^−^: Chloride
Cu^2^^+^: Copper
CV: Coefficient of variation
ENaC: Epithelial sodium channel
Fe^2^^+^: Iron
GLUT: Glucose transporter
H^+^: Hydrogen
IK: Intermediate conductance Ca^2^^+^-activated K^+^ channel
IL: Interleukin
K^+^: Potassium
Mg^2^^+^: Magnesium
MCT: Monocarboxylate transport
mRNA: Messenger ribonucleic acid
Na^+^: Sodium
NHE: Na^+^/H^+^ exchanger
NH_3_: Ammonia
NMF: Natural moisturizing factors
pKa: Negative log of the acid dissociation constant
SGLT: Sodium-dependent glucose transporter
SLC: Solute carrier
TGF: Transforming growth factor
TNF: Tumor necrosis factor
TMEM16A: Transmembrane member 16A
TRPC: Transient receptor potential channel
TRPV1: Transient receptor potential cation channel subfamily V member 1
Zn^2^^+^: Zinc
[]: Concentration
