# Supplementary material for: Physiological mechanisms determining eccrine sweat composition
Source: Eur J Appl Physiol. 2020 Mar 2;120(4):719–52. doi: 10.1007/s00421-020-04323-7 (PMC7125257; doi:10.1007/s00421-020-04323-7)
Supplement: Supplementary file 1 — Supplementary file1 (DOCX 15 kb) [file 421_2020_4323_MOESM1_ESM.docx]

**Supplemental Table 1**: Reference list for sweat constituent concentrations in Table 1

| **Sweat constituent** | **References** |
| --- | --- |
| Sodium and/or Chloride | Maughan and Shirreffs 1998; Baker et al. 2009; Patterson et al. 2000; Verde et al. 1982; Baker et al. 2016: Baker et al. 2018; Johnson and Pitts, 1944; Baker et al. 2019; Dill et al. 1966 |
| Lactate | Patterson et al. 2000; Sato 1977; Weiner and Van Heyningen 1952; Buono et al. 2010; Green et al. 2000; Fellmann et al. 1983; Alvear-Ordenes et al. 2005 |
| Urea | Alvear-Ordenes et al. 2005; Amatruda and Welt 1953; Weiner and Heyningen 1952 |
| Potassium | Patterson et al. 2000; Baker et al. 2009 |
| Ammonia | Alvear-Ordenes et al. 2005; Amatruda and Welt 1953; Ament et al. 1953; Sato et al. 1989 |
| Ethanol | Buono 1999; Phillips and McAloon 1980 |
| Bicarbonate | Patterson et al. 2000; Patterson et al. 2002 |
| Trace Minerals (Ca, Mg, Fe, Cu, Zn) | Ely et al. 2011, Baker et al. 2011, Montain et al. 2007; Shirreffs and Maughan 1997; Chinevere 2008; Ely et al. 2013; Costa, 1969; Paulev et al. 1983; Brune et al. 1986; Aruoma et al. 1988; Cohn and Emmett 1978 |
| Glucose | Moyer et al. 2012; Boysen et al. 1984; Lee et al. 2017 |
| Amino acids | Mark and Harding 2013 |
| Vitamins (thiamine and ascorbic acid) | Thapar et al. 1976, Mickelsen and Keys 1943 |
| Cortisol | Jia et al. 2016 ; Russell et al. 2014 |
| Cytokines (IL-1 alpha, IL-1 beta, TGF beta, IL-6, IL-8, TNF alpha) | Marques-Deak et al. 2006; Cizza et al. 2008; Hladek et al. 2018; Jones et al. 1995; Katchman et al. 2018 |
