# Supplementary material for: Physiological mechanisms determining eccrine sweat composition
Source: Eur J Appl Physiol. 2020 Mar 2;120(4):719–52. doi: 10.1007/s00421-020-04323-7 (PMC7125257; doi:10.1007/s00421-020-04323-7)
Supplement: Supplementary file 2 — Supplementary file2 (DOCX 21 kb) [file 421_2020_4323_MOESM2_ESM.docx]

Supplemental Table 2. Summary of studies comparing sweat constituent concentrations of sweat with and without epidermal contamination.

| **Reference** | **Methods** | **Constituent** | **Results** | **Effect of epidermal contamination on sweat constituent concentrations** |
| --- | --- | --- | --- | --- |
| Boysen et al. 1984 | Anaerobic method (pouch with Vaseline/mineral oil) vs. collection pouch w/o Vaseline/mineral oil vs. scraped sweat; sweating induced via sauna (45-60°C, 70% rh); sweat collected from upper back after an initial 10 min in sauna and every 5 min thereafter for a total of 40 min.  No differences in local sweating rate between anaerobic method and collection pouch w/o oil. Scraped sweating rate data NA. | Sodium | No difference in [Na] among methods. | ↔ |
| Ely et al. 2011 | Sweat collection from the arm (bag method) and upper back (pouch method) in the first 20-30 min of each hour during 3 h treadmill walking (35°C, 20% rh) in 16 participants (14 men, 2 women). Skin was meticulously cleaned at both sites. Only cell-free sweat was analyzed.  Local sweat rate not standardized between the arm bag and pouch.  Arm bag: 0.17, 0.29, and 0.29 L/m^2^/h in the 1^st^, 2^nd^, and 3^rd^ h, respectively  Upper back pouch: 0.51, 0.43, 0.30 L/m^2^/h in the 1st, 2nd, and 3rd h, respectively | Sodium | No skin surface contamination found. | ↔ |
| Boysen et al. 1984 | See Boysen et al. methods above | Potassium | No difference in [K] between anaerobic method and collection pouch w/o oil. Sweat [K] 30-40% higher with scraping vs. anaerobic method. | ↑ by 30-40% |
| Ely et al. 2011 | See Ely et al. methods above | Potassium | Sweat [K] was ~2 to 3-fold higher in the arm bag (~12-16 mmol/L) vs. sweat pouch (5.25 mmol/L) throughout 3 h exercise. | ↑ by ~2 to 3-fold* |
| Adams et al. 1950 | Sweat collected in arm bag during 1-h water bath (43°C) in 25 participants. Both cell-rich and cell-free sweat was analyzed. | Iron | Cell-rich sweat: 7.06 µg/ml  Cell-free sweat: 0.30 µg/ml | ↑ by 24-fold |
| Foy and Kondi, 1957 | Whole body sweat collected in a thermostatically controlled room in adults. Both cell-rich and cell-free sweat was analyzed. | Iron | Cell-rich sweat: 0.3-6.0 mg/L  Cell-free sweat: 0.1-0.2 mg/L | ↑ by 3 to 40-fold |
| Hussain and Patwardhan, 1959 | Sweat collected in arm bag during 1-h water bath (42-43°C) in 6 women. Both cell-rich and cell-free sweat was analyzed. | Iron | Cell-rich sweat: 1.61 mg/L  Cell-free sweat: 0.44 mg/L | ↑ by 3.7-fold |
| Hussain et al. 1960 | Sweat collected in arm bag during 1-h thermal sweating in 35 men. Both cell-rich and cell-free sweat was analyzed. | Iron | Cell-rich sweat: 1.15 mg/L  Cell-free sweat: 0.34 mg/L | ↑ by 3.4-fold |
| Apte and Venkatachalam, 1962 | Sweat collected from different body regions with various methods (plastic capsules, sweat bags, or filter paper discs) during 20-60 min thermal sweating in 6 to 16 men across 3 experiments. Both cell-rich and cell-free sweat was analyzed. | Iron | Iron concentration of cell free sweat was significantly lower than that of the cell rich sweat  Cell-rich sweat:  Upper extremity: 330 µg/L  Lower extremity: 520 µg/L  Hot season: 356 µg/L  Collection at 20 min: 272 µg/L  Collection at 40 min: 342 µg/L  Cell-free sweat:  Upper extremity: 190 µg/L  Lower extremity: 250 µg/L  Hot season: 190 µg/L  Collection at 20 min: 219 µg/L  Collection at 40 min: 197 µg/L | ↑ by 1.2 to 2.1 fold |
| Prasad et al. 1963 | Sweat collected in arm bag during 2-3 h passive heat (sun) exposure (38-41°C) in 8-10 healthy men. Both cell-rich and cell-free sweat was analyzed. | Iron | Cell-rich sweat: 1.20 µg/ml  Cell-free sweat: 0.46 µg/ml | ↑ by 2.6-fold |
| Vellar, 1968 | Whole body sweat collected during 60-min passive heat stress (40-45°C, 80-90% rh) in 23 men. Both cell-rich and cell-free sweat was analyzed. | Iron | Cell-rich sweat: 41.2 µg/100 ml  Cell-free sweat: 29.8 µg/100 ml | ↑ by 1.4-fold |
| Paulev et al. 1983 | Group 1: Sweat collected from back of endurance athletes (n=10) directly into test tubes during first 10 min of cycling (21°C) without previous washing of skin  Group 2: Sweat collected from back of endurance athletes (n=10) directly into test tubes; collection began after an initial 10 min of cycling (21°C) and again every ~10 min thereafter for a total of ~30 min.  Sweating Rate data NA | Iron | Group 1:  1^st^ sample (0-10 min of exercise): 5.2 µmol/L  Group 2:  1^st^ sample: discarded  2^nd^ sample (10-20 min of exercise): 3.6 µmol/L  3^rd^ sample (21-30 min of exercise): 2.3 µmol/L  4^th^ sample (31-38 min of exercise): 2.4 µmol/L  More skin cells were found by microscopy in the 2^nd^ vs. the 4^th^ sample, thus the 4^th^ sample was deemed relatively cell-poor and the 2^nd^ sample relatively cell-rich | ↑ by up to 2.2-fold |
| Brune et al. 1986 | Whole body sweat collected during sauna exposure (~41°C, ~87% rh) in 11 men. Participants had extensive skin cleaning and were exposed to 10 min in sauna prior to sweat collection/analysis. Two 30 min sweat collection periods commenced thereafter. Both cell-rich and cell-free (supernatant after centrifuging) sweat was analyzed.  In a subset of participants (n=2), sweat was collected/analyzed for four 30-min periods.  Whole body sweat loss was not different between the 1st (583 ml) and 2nd (581 ml) periods of sweat collection | Iron | Cell-rich sweat:  1^st^ sample: 213 µg/L  2^nd^ sample: 119 µg/L  Cell-free sweat:  1^st^ sample: 50.7 µg/L  2^nd^ sample: 22.5 µg/L  In the sub-study with 4 collection periods, sweat [Fe] was consistent from the 2^nd^ sample onwards | ↑ by up to 5.3-fold |
| Ely et al. 2011 | See Ely et al. methods above | Iron | Sweat [Fe] was 7 to 9-fold higher in the arm bag (~2-2.5 µmol/L) vs. sweat pouch (0.28 µmol/L) throughout 3 h exercise. | ↑ by ~7 to 9-fold* |
| Boysen et al. 1984 | See Boysen et al. methods above | Calcium | In the 10-min sample, sweat [Ca] was 6-fold higher in scraped sweat and 2.5-fold higher in collection pouch w/o oil compared with anaerobic method. Gradual lessening of difference among methods over time. Sweat [Ca] similar among methods after 25-30 min of profuse sweating. | ↑ by up to 2.5 to 6-fold |
| Ely et al. 2011 | See Ely et al. methods above | Calcium | Sweat [Ca] was ~4-fold higher in the arm bag (~1.6 mmol/L) vs. sweat pouch (0.36 mmol/L) in the first hour of exercise; no differences in hour 2-3. | ↑ by ~4-fold* |
| Ely et al. 2011 | See Ely et al. methods above | Magnesium | Sweat [Mg] was ~6-fold higher in the arm bag (~0.3 mmol/L) vs. sweat pouch (0.05 mmol/L) in the first hour of exercise; no differences in hour 2-3. | ↑ by ~6-fold* |
| Ely et al. 2011 | See Ely et al. methods above | Copper | Sweat [Cu] was ~3 to 10-fold higher in the arm bag (~2-7 µmol/L) vs. sweat pouch (0.72 µmol/L) in the first 2 h of exercise; no difference in hour 3. | ↑ by up to ~3 to 10-fold* |
| Prasad et al. 1963 | See Prasad et al. methods above | Zinc | Cell-rich sweat: 1.15 µg/ml  Cell-free sweat: 0.93 µg/ml | ↑ by 1.2-fold |
| Ely et al. 2011 | See Ely et al. methods above | Zinc | Sweat [Zn] was ~2.5-fold higher in the arm bag (~10 µmol/L) vs. sweat pouch (3.64 µmol/L) in the first hour of exercise; no differences in hour 2-3. | ↑ by ~2.5-fold* |
| Boysen et al. 1984 | See Boysen et al. methods above | Protein | In the 10-min sample, sweat [protein] was ~2.7-fold higher in scraped sweat and ~1.6-fold higher in collection pouch w/o oil compared with anaerobic method. Sweat [protein] similar between anaerobic and pouch w/o oil method after 15-20 min profuse sweating. Scraped sweat [protein] gradually decreased over time but remained elevated (by ~1.5 fold) above other methods. | ↑ by up to ~1.6 to 2.7-fold* |
| Boysen et al. 1984 | See Boysen et al. methods above | Glucose | No difference in [Glucose] among methods. | ↔ |
| Boysen et al. 1984 | See Boysen et al. methods above | Lactate | No difference in [Lactate] between anaerobic method and collection pouch w/o oil. Sweat [Lactate] 30-40% higher with scraping vs. anaerobic method. | ↑ by 30-40% |
| Boysen et al. 1984 | See Boysen et al. methods above | Cholesterol | No difference in [Chol] between anaerobic method and collection pouch w/o oil. Sweat [Chol] ~5.5-fold higher with scraping vs. anaerobic method. | ↑ by ~5.5-fold* |
| Boysen et al. 1984 | See Boysen et al. methods above | cAMP | No difference in [cAMP] between anaerobic method and collection pouch w/o oil. Sweat [cAMP] ~3 to 5-fold higher with scraping vs. anaerobic method. | ↑ by ~3 to 5-fold* |
| Boysen et al. 1984 | See Boysen et al. methods above | Urea | In the 10-min sample, sweat [urea] was ~2.4-fold higher in scraped sweat and ~1.4-fold higher in collection pouch w/o oil compared with anaerobic method. Gradual lessening of difference among methods over time. Sweat [urea] similar among methods after 25-30 min of profuse sweating. | ↑ by up to ~1.4 to 2.4-fold* |
| Brusilow and Ikai, 1968 | Anaerobic method vs. filter paper on contralateral arms (anterior surface); pilocarpine iontophoresis; sweat collected 30 min after sweat stimulation  No difference in local sweat secretory rates between methods. | Urocanic Acid | Anerobic method: 0.76 mg/100ml  Filter paper: 5.4 mg/100 ml | ↑ by 7-fold |

Sweat constituent concentration, sweat loss, and sweating rate values are mean data reported in original papers. *Differences estimated from published figures.
