# Supplementary material for: Physiological mechanisms determining eccrine sweat composition
Source: Eur J Appl Physiol. 2020 Mar 2;120(4):719–52. doi: 10.1007/s00421-020-04323-7 (PMC7125257; doi:10.1007/s00421-020-04323-7)
Supplement: Supplementary file 3 — Supplementary file3 (DOCX 27 kb) [file 421_2020_4323_MOESM3_ESM.docx]

Supplemental Table 3. Summary of studies comparing the effect of various methods of sweat stimulation on sweat constituent concentrations.

| **Reference** | **Methods** | **Constituent** | **Results** | **Difference in sweat constituent concentration between methods^†‡^** |
| --- | --- | --- | --- | --- |
| Shwachman and Antonowicz 1962 | Sweat was collected from the left forearm passively (using a bag technique for ≤ 1 h with no external heat) or after pilocarpine iontophoresis in 17 healthy controls and 13 cystic fibrosis patients. Sweating rate data NA. | Sodium and Chloride | Stats NA, but similar [Na] and [Cl] values reported for passive (bag) sweat and pilocarpine sweat in controls (Na: 17 vs. 20 mEq/L; Cl: 18 vs. 16 mEq/L). Bag sweat marginally higher than pilocarpine sweat in CF patients (Na: 104 vs. 94 mEq/L; Cl: 114 vs. 107 mEq/L). | Passive Heat ≥ Pharmacological |
| Di Sant’Agnese and Powell 1962 | Sweat was collected with gauze pads during thermal stimulation at constant room temp (abdomen or scapula, n=355-938) or pilocarpine iontophoresis (forearm, n=84-166) in pediatric cystic fibrosis patients and healthy controls. Sweating rate data NA. | Sodium and Chloride | No difference in [Na] or [Cl] between thermal sweat and pilocarpine sweat in CF patients (Na: 104 vs. 101 mEq/L; Cl: 97 vs. 99 mEq/L) or controls (Na: 21 vs. 23 mEq/L; Cl: 19 vs. 16 mEq/L). | Passive Heat = Pharmacological |
| Collins 1962 | Sweat was collected from the palm and forearm of 6 participants using the sweat capsule method during indirect heating (legs in a water bath 43-45°C) or intradermal injection of methacholine (30 min). Sweat collections were done at 20 min intervals for 1 h with indirect heating and 10 min intervals for 30 min after methacholine injection. Local sweating rate was not matched between methods (stats NA). Sweating rate was higher with methacholine vs. indirect heating trials on the palm (11-23 vs. 5 mg/10cm^2^/10min). On the forearm sweating rate was higher with methacholine vs. indirect heating only for the first 20 min (40-50 vs. 9 mg/10cm^2^/10min). Thereafter forearm sweating rate was higher with indirect heating (66-87 mg/10cm^2^/10min from 20-60 min) vs. methacholine (37 mg/10cm^2^/10min from 20-30 min) | Sodium | Stats NA, but trends for higher sweat [Na] means with methacholine vs. indirect heating trials  Forearm  Passive Heat^#^: 19, 26, and 28 mEq/L at 0-20, 20-40, and 40-60 min, respectively.  Methacholine^#^: 61, 43, and 37 mEq/L at 0-10, 10-20, and 20-30 min, respectively.  Palm  Passive Heat^#^: 22, 24, and 16 mEq/L at 0-20, 20-40, and 40-60 min, respectively.  Methacholine^#^: 52, 34, and 23 mEq/L at 0-10, 10-20, and 20-30 min, respectively. | Pharmacological > Passive Heat |
| Collins 1962 | See Collins methods above | Chloride | Stats NA, but trends for higher sweat [Cl] means with methacholine vs. indirect heating trials  Forearm  Passive Heat^#^: 18, 22, and 29 mEq/L at 0-20, 20-40, and 40-60 min, respectively.  Methacholine^#^: 38, 29, and 28 mEq/L at 0-10, 10-20, and 20-30 min, respectively.  Palm  Passive Heat^#^: 16, 20, and 12 mEq/L at 0-20, 20-40, and 40-60 min, respectively.  Methacholine^#^: 38, 33, and 20 mEq/L at 0-10, 10-20, and 20-30 min, respectively. | Pharmacological > Passive Heat |
| Kozlowski and Saltin 1964 | Sweat was collected from 6 participants with the arm bag technique during 3 h passive heat (sauna 80°C, 15% rh), exercise (18°C, 50% rh), or exercise+heat (38°C, 35% rh) where sweating rate was matched among conditions. | Sodium and Chloride | Trend for higher sweat [Na] (86 vs. 77 and 66 mEq/L) and [Cl] (67 vs 65 and 57 mEq/L) with passive heat stress vs. exercise and exercise +heat (Stats NA).* | Passive Heat ≥ Exercise = Exercise+Heat |
| Ikai et al. 1969 | Sweat was collected from the back of 23 participants (men and women) with a gauze pad following passive (heat stress (38-40°C) or pilocarpine iontophoresis) or exercise-induced sweating. Sweating rate was 3.6 g/100cm^2^ with passive heat and 2.7 g/100cm^2^ with exercise (stats NA). | Sodium and Chloride | Sweat [Na] (76 vs. 55 mEq/L) and [Cl] (65 vs. 47 mEq/L) significantly higher (by 1.4-fold) with exercise vs. passive heat stress. | Exercise > Passive Heat /Pharmacological |
| Sato et al. 1970 | Sweat was collected from the volar forearm of 15 men with filter paper after pharmacological stimulation (pilocarpine iontophoresis or intracutaneous injection of pilocarpine, methylcholine, and acetylcholine) or passive heat stress (44-47°C, 80% rh). Sweating rate data NA. | Sodium | Higher sweat [Na] with pharmacological stimulation vs. passive heat stress (mean values NA). | Pharmacological > Passive Heat |
| Verde et al. 1982 | Sweat was collected from the forearm of 8 men with gauze pads during exercise (outdoor running; 14-23°C) or sauna exposure (93°C) where sweating rate was matched between conditions. | Sodium and Chloride | No difference in sweat [Na] (61 vs. 59 mEq/L) or [Cl] (46 vs. 39 mEq/L) between sauna and exercise. | Passive Heat = Exercise |
| Fukumoto et al. 1988 | Sweat was collected from the chest of 7 men by scooping it into a test tube after passive heat stress (40°C, 60% rh) or running exercise. Sweating rate data NA. | Sodium and Chloride | Sweat [Na] and [Cl] significantly higher (by 1.5 to 1.6-fold) with passive heat stress vs. exercise. | Passive Heat > Exercise |
| Shwachman and Antonowicz 1962 | Sweat was collected from the left forearm passively (using a bag technique for ≤ 1 h with no external heat) or after pilocarpine iontophoresis in 17 healthy controls and 13 cystic fibrosis patients. Sweating rate data NA. | Potassium | Stats NA, but [K] values reported for pilocarpine sweat were higher than passive (bag) sweat in controls (10 vs. 7 mEq/L) and CF patients (16 vs. 10 mEq/L). | Pharmacological > Passive Heat |
| Collins 1962 | See Collins methods above | Potassium | Stats NA and no clear trends for a difference in sweat [K] means between methacholine and indirect heating trials  Forearm  Passive Heat^#^: 7, 4, and 4 mEq/L at 0-20, 20-40, and 40-60 min, respectively.  Methacholine^#^: 7, 6, and 7 mEq/L at 0-10, 10-20, and 20-30 min, respectively.  Palm  Passive Heat^#^: 25, 19, and 12 mEq/L at 0-20, 20-40, and 40-60 min, respectively.  Methacholine^#^: 21, 15, and 13 mEq/L at 0-10, 10-20, and 20-30 min, respectively. | Pharmacological=Passive Heat |
| Kozlowski and Saltin 1964 | See Kozlowski and Saltin methods above | Potassium | Trend for higher sweat [K] with exercise (12.7 mEq/L) vs. heat stress (11.3 mEq/L) and exercise+heat (10.3 mEq/L) (Stats NA).* | Exercise ≥ Passive Heat = Exercise+Heat |
| Ikai et al. 1969 | See Ikai et al. methods above | Potassium | No difference in sweat [K] (3.7 mEq/L) between conditions. | Exercise = Passive Heat/Pharmacological |
| Sato et al. 1970 | See Sato et al. methods above | Potassium | Higher sweat [K] (by 1.7-fold) in all participants with pharmacological stimulation (8.3 mEq/L) vs. passive heating (4.9 mEq/L). | Pharmacological > Passive Heat |
| Verde et al. 1982 | See Verde et al. methods above | Potassium | No difference in sweat [K] (7.9 vs. 9.4 mEq/L) between sauna and exercise. | Passive Heat = Exercise |
| Fukumoto et al. 1988 | See Fukumoto et al. methods above | Potassium | Sweat [K] significantly higher (by 1.3-fold) with exercise vs. passive heat stress. | Exercise > Passive Heat |
| Verde et al. 1982 | See Verde et al. methods above | Calcium and Magnesium | Sweat [Ca] (4.7 vs. 2.7 mEq/L) and [Mg] (1.8 vs. 0.8 mEq/L) significantly higher (by 1.7 and 2.3-fold, respectively) with sauna vs. exercise. | Passive Heat > Exercise |
| Collins 1962 | See Collins methods above | Lactate | Stats NA. No trend for difference in forearm sweat [Lactate] between methods, but trend for higher palm sweat [Lactate] with indirect heating vs. methacholine trials.  Forearm  Passive Heat^#^: 23, 16, and 15 mEq/L at 0-20, 20-40, and 40-60 min, respectively.  Methacholine^#^: 20, 15, and 14 mEq/L at 0-10, 10-20, and 20-30 min, respectively.  Palm  Passive Heat^#^: 67, 64, and 44 mEq/L at 0-20, 20-40, and 40-60 min, respectively.  Methacholine^#^: 48, 29, and 22 mEq/L at 0-10, 10-20, and 20-30 min, respectively. | Passive Heat ≥ Pharmacological |
| Astrand 1963 | Sweat was collected from the forearms or legs of 4 participants (2 men, 2 women) via a plastic bag method during cycling exercise or water bath (19.0-45.3°C). Sweat was collected in 15 min intervals for 60 min. Sweating rate ranged from ~0.02-1.1 g/min. It was not standardized between conditions and was generally higher with bathing. | Lactate | At each time point sweat lactate concentrations were higher with cycling than bathing. Sweat [lactate] decreased over time when cycling, but did not change over time with bathing.  Cycling: 338, 195, 165, and 156 mg/100 ml during the 1^st^, 2^nd^, 3^rd^, and 4^th^ 15-min periods, respectively.  Bathing: 224, 175, and 137 mg/100 ml during the 1^st^, 2^nd^, and 3^rd^ 15-min periods, respectively. | Exercise > Passive Heat |
| Mitsubayashi et al. 1994 | Sweat was collected from the upper body of 4 healthy untrained participants via a scraping method during a 5-min hot bath (45°C), exhaustive exercise (stair sprinting, <25°C), and continuous submaximal exercise (3000 m run, <25°C). Sweating rate data NA. | Lactate and Ammonium | Sweat lactate concentration was 2.0 and 5.2-fold higher during exhaustive and continuous exercise, respectively, versus passive heating.  Sweat ammonia concentration was 3.0 and 10.6-fold higher during exhaustive and continuous exercise, respectively, versus passive heating. | Exercise > Passive Heat |
| Fukumoto et al. 1988 | See Fukumoto et al. methods above | Creatinine and Urea Nitrogen | Concentrations of creatinine and urea nitrogen significantly higher (by 1.7 and 2.4-fold, respectively) with exercise vs. passive heat stress. | Exercise > Passive Heat |
| Liappis and Hungerland 1972 | Sweat was collected from the face and abdomen after a 15-min water bath (n=2; 40-41°C). Sweat was also collected from the same regions during exercise (n=7). Sweating rate data NA. | Amino Acids | Sweat amino acid concentrations were significantly higher during exercise than at rest. In general, amino acids concentrations were typically ~3 fold higher with exercise. Arginine, citrulline, and cysteine were 18, 2, and 1.2-fold higher, respectively, with exercise than rest. | Exercise > Passive Heat |
| Souza et al. 2018 | Sweat was collected from the forearm of 20 participants (men and women) with filter paper during natural sweating (7 h), pilocarpine iontophoresis, or exercise (20 min). Sweating rate was significantly higher with pilocarpine and exercise vs. natural passive sweating. | 34 Metabolites and Amino acids | Concentrations of most metabolites and amino acids were significantly higher (by 3 to 7-fold) in natural sweat than sweat induced via pilocarpine and physical exercise. Concentrations of some constituents (alanine, aspartate, asparagine, isoleucine, leucine, lysine, pyruvate, and valine) significantly higher (by 1.1 to 4.0-fold) in sweat induced via exercise vs. pilocarpine iontophoresis. | Passive > Exercise ≥ Pharmacological |
| Agrawal et al. 2018 | Sweat was collected from the volar forearm of 7 men via a Macroduct sweat collector following pilocarpine iontophoresis or exercise (15 min cycling; 23°C, 35% rh). Sweating rate data NA. | 70 Lipid Mediators | Concentrations of most lipid mediators (alcohols, diols, epoxides, ketones, triols, monoacyglycerols, aceylglycines, and sphingolipids) significantly higher (by 2 to 9-fold) in pilocarpine vs. exercise sweat. | Pharmacological > Exercise |
| Delgado-Povedano et al. 2018 | Sweat was collected from the head, back, and chest of 6 participants (4 men and 2 women) after 30 min of exercise using a micropipette. Passive sweat was also collected from the forearm using a Macroduct after administration of pilocarpine iontophoresis. Sweating rate data NA. | Alcohols, Carbohydrates, and Fatty Acids | All measured fatty acids (except caproic and stearic acid), carbohydrates (monosaccharides and sugar alcohols), alcohols (diols, glycols), and non-proteinogenic amino acids were significantly higher in exercise-induced sweat than pilocarpine-induced sweat. | Exercise > Pharmacological |
| Talbert 1919 | Sweat was collected into specimen tubes from the face, chest, and abdomen from participants (n=9, total of 53 observations) during 15-25 min passive heating (30-45°C) or 15-25 min cycling exercise. Sweating rate data NA. | pH | Sweat pH was higher with exercise (6.22) than passive heating (5.73). | Exercise > Passive Heat |
| Talbert 1922 | Same methods as Talbert 1919, with the addition of a rubber jacket to better control for the effect of ventilation and local skin humidity (total of 46 observations). Sweating rate data NA. | pH | Sweat pH was not different between exercise (5.65) and passive heating (5.64). | Passive Heat = Exercise |
| Didierjean et al. 1990 | Sweat was collected from the hand using the anaerobic technique in combination with a petroleum barrier after stimulation by pilocarpine iontophoresis (n=4) or sauna bathing (n=6) or spontaneous sweating (n=11). Authors reported that sweating rate was highest with sauna, but specific data NA. | Cytokines (IL-1) | Sweat IL-1α concentration was higher with passive heating (22.67 ng/mL) than pilocarpine (3.97 ng/mL) and spontaneous sweating (3.72 ng/mL). No differences between pilocarpine and spontaneous sweat.  Sweat IL-1β concentration was higher with passive heating (3.36 ng/mL) than pilocarpine (1.23 ng/mL) and spontaneous sweating (0.33 ng/mL). Sweat IL-1β concentration was also higher in pilocarpine than spontaneous sweat. | IL-1α: Passive Heat > Pharmacological = Passive  IL-1β: Passive Heat > Pharmacological > Passive |

Sweat concentration values are mean data reported in original papers (rounded to nearest whole number for sodium and chloride). ^#^Means calculated from individual data. *Sweat electrolyte concentrations calculated from body water loss (kg) and sweat electrolyte loss (mEq) data reported in Kozlowski and Saltin 1964. †Passive Heat = sweating via thermal stimulation (e.g., sauna, dry heat, or water bath). ‡Passive = natural, spontaneous sweating without specific means of intentional stimulation.
