# Supplementary material for: Physiological mechanisms determining eccrine sweat composition
Source: Eur J Appl Physiol. 2020 Mar 2;120(4):719–52. doi: 10.1007/s00421-020-04323-7 (PMC7125257; doi:10.1007/s00421-020-04323-7)
Supplement: Supplementary file 4 — Supplementary file4 (DOCX 29 kb) [file 421_2020_4323_MOESM4_ESM.docx]

Supplemental Table 4. Summary of studies comparing sweat and blood constituent concentrations

| **Reference** | **Constituent** | **Methods** | **Results** | **Significant Correlation?** |
| --- | --- | --- | --- | --- |
| Hew-Butler et al. 2010 | Sodium | 10 moderately-trained participants (men and women) completed a maximal test to exhaustion and 60-min steady state run. Serum [Na] was measured pre and post-run. Sweat [Na] was measured from sweat collected with gauze pads on the back during exercise. | Significant correlation (r=0.64, p<0.01) between sweat and post-exercise serum [Na] when data from the maximal test (n=6) and steady state (n=10) runs were combined. Correlation results not reported for the pre-exercise serum [Na] or within each type of exercise. | Unclear |
| McCubbin et al. 2019 | Sodium | 15 male endurance athletes consumed a low, high, or their usual Na diet for 3 days. On day 4 they completed 2 h running or cycling exercise (35°C). Sweat [Na] was measured in samples collected from 5 sites via absorbent patches during the 1^st^ and 2^nd^ hour of exercise. Plasma [Na] was measured pre- and post-exercise. Whole body sweat [Na] was predicted from previously published regression equations. | No significant correlation between pre-exercise plasma [Na] and whole-body sweat [Na] in the 1^st^ hour (r=0.18, p>0.05) or 2^nd^ hour (r=0.21, p>0.05) of exercise. | No |
| Talbert and Haugen 1927 | Chloride | Sweat [Cl] and blood [Cl] were measured in 17 men 1-12x each during 30-45 min exposure to the heat or physical work. | Authors noted evidence of a correlation (r=0.64, p value NA) between blood [Cl] and sweat [Cl], with the omission of two extreme outliers. Blood [Cl] range was 3.3 to 3.9 mg/ml. Sweat [Cl] range was 4.3 to 8.3 mg/ml. | Unclear |
| Mickelsen and Keys 1943 | Chloride | Sweat [Cl] (arm bag technique) and plasma [Cl] were measured in 18 men during exposure to the heat (49°C, 25% rh). | Authors concluded that there was no correlation between serum [Cl] and sweat [Cl] (figure shown, but r value not reported). | No |
| Johnson et al. 1944 | Chloride | 2-3 men marched 13 miles on a treadmill (3.5 mph, 2.5% grade; 38°C, 35% rh) while serum [Cl] and sweat [Cl] (arm bag technique) were measured at 1-h and post-exercise time points. In separate experiments, water or saline (n=3) and water or no fluid (n=2) was consumed during the march. | Authors concluded that there was no correlation between serum [Cl] and sweat [Cl] (r value not reported).  Experiment 1  1h: sweat [Cl] 83 mEq/L with water and 72 mEq/L with saline; serum [Cl] 102 mEq/L with water and 101 mEq/L with saline  4h: sweat [Cl] 96 mEq/L with water and 79 mEq/L with saline; serum [Cl] 98 mEq/L with water and 99 mEq/L with saline  Experiment 2  1h: sweat [Cl] 44 mEq/L with water and 48 mEq/L without water; serum [Cl] 104 mEq/L with water and 107 mEq/L without water  3h: sweat [Cl] 49 mEq/L with water and 58 mEq/L without water; serum [Cl] 103 mEq/L with water and 108 mEq/L without water | No |
| Robinson et al. 1956 | Chloride | 4 men completed 25-h intermittent exercise+heat protocols (44°C/26°C) 4 times (differing in the amount and NaCl content of fluid intake). Serum [Cl] and sweat [Cl] (whole body technique) were measured at nine different time points during the 25-h protocol. | Authors stated that there was a positive relation between serum [Cl] and sweat [Cl] in 3 of 4 participants at the 25-h time point (end of study) only, but r value and mean group data not reported. | Unclear |
| Vellar, 1968 | Iron | Sweat [Fe] measured (whole body technique) during 60-min passive heat stress (40-45°C, 80-90% rh) in 23 men. Both cell-rich and cell-free sweat were analyzed. Serum [Fe] was measured PRE and POST sweat collection. | Cell-rich sweat  No significant correlation between PRE serum [Fe] and sweat [Fe] (r=0.29, p>0.05)  No significant correlation between POST serum [Fe] and sweat [Fe] (r=0.25, p>0.05)  Cell-free sweat  No significant correlation between PRE serum [Fe] and sweat [Fe] (r=0.25, p>0.05)  Significant correlation between POST serum [Fe] and sweat [Fe] (r=0.37, 0.05<p<0.1) | Unclear |
| Paulev et al. 1983 | Iron | Serum [Fe] was measured at baseline and sweat [Fe] (from the back directly into test tubes) was measured during 10-30 min of cycling exercise (21°C) in 20 endurance athletes. | No correlation (r=0.03, p>0.05) between serum [Fe] and iron [Fe] | No |
| Mickelsen and Keys 1943 | Ascorbic Acid | Experiment 1  12 men maintained fixed intakes of ascorbic acid (25 and 525 mg/day) for 4-7 days. Sweat (arm bag technique) was measured during passive heat stress (49°C, 25%) or treadmill exercise.  Experiment 2  6 men ingested 50 mg of pure ascorbic acid. Sweat (arm bag technique) and plasma ascorbic acid concentrations were measured before and 2 and 6 h post-ingestion. | Experiment 1  Concentration of ascorbic acid in sweat was low and did not reflect the difference in dietary intake (r value not reported). Sweat ascorbic acid concentration was 0.059 mg/dl with 525 mg/day intake and 0.060 mg/dl with 25 mg/day intake.  Experiment 2  Concentration of ascorbic acid in sweat was independent of that in the plasma (which ranged from 0.31 to 1.41 mg/dl) (r value not reported). Sweat ascorbic acid concentration was 0.028 mg/dl before ingestion, 0.27 mg/dl 2 h after ingestion, and 0.035 mg/dl 6 h after ingestion. | No |
| Silvers et al. 1928 | Glucose | Sweat was collected from 11 participants during passive heating. Blood was collected immediately before and after sweating. | Authors conclude that there was no correlation between sweat and blood glucose concentrations. Correlation data not reported, but r was -0.09 to 0.01 based on calculations from raw data listed in the paper. | No |
| Boysen et al. 1984 | Glucose | Sweat was collected from the upper back of 2 participants using an anaerobic technique with an oil barrier during 50 min in a sauna (50°C, 70% rh). After 20 min in the sauna, participants ingested 100 g glucose and were administered 25 g glucose via intravenous infusion. Sweat and blood were collected at baseline and every 5 min for a total of 30 min after the glucose bolus. | Plasma glucose concentration increased from 60 to 360 mg/dl after the glucose bolus. Sweat glucose concentration significantly increased in both participants concurrent with the increase in plasma glucose. Correlation results (r values) were not reported. | Unclear |
| Moyer et al. 2012 | Glucose | 7 diabetic patients were administered glucose or insulin to induce a change in blood glucose (full range was ~60-360 mg/dl across all trials). Sweat was collected from the right and left forearms at 10-min intervals using a perfusion device after stimulation by pilocarpine iontophoresis. A barrier substance (petroleum and paraffin) was applied to the skin after sweat stimulation. Capillary blood samples were also collected at 10-min intervals. | Mean correlation between sweat and blood glucose concentrations across 23 separate trials (2 arms x 12 trials per participant, minus 1 trial because of missing data) was r=0.83 (p value NA and individual results not reported). All sweat glucose values were adjusted for time lag. | Yes |
| Lee et al. 2016 | Glucose | In 2 participants, sweat was collected/analyzed with a novel graphene-based electrochemical patch. Method of sweat stimulation unclear. Sweat and blood samples (~90-140 mg/dl) were collected hourly from 800h to 2200h. | r^2^=0.83 (p<0.001) reported for 15 data points, but it is unclear if these data points include an aggregate of both participants or just one of the participants (since there were 15 time points per participant) | Yes |
| Lee et al. 2017 | Glucose | In 5 participants, blood was collected before and after a meal (~4.5-8 mmol/L). After each blood sample, sweat was collected/analyzed via a novel electrochemical patch during cycling exercise. | r=0.76 (p value NA) reported for 20 samples, but unclear what time points or participants were included. r=0.91 and r=0.87 reported for individual participants (6 data points per participant), but unclear why only 2 of 5 participants individual data were reported. | Unclear |
| Bandodkar et al. 2019 | Glucose | See Bandodkar et al. methods above | Authors conclude that there is “some potential” for using sweat to track blood glucose, but r value NA in main paper and supplemental material not available from the journal. | Unclear |
| Nyein et al. 2019 | Glucose | In 20 healthy and 28 diabetic participants, blood (glucose concentration was 80-250 mg/dl across all participants) and sweat samples were collected. Sweat was collected with novel microfluidic-based patches during exercise for up to 90 min and during pilocarpine iontophoresis for 28-32 min. | There was a significant correlation between sweat and blood glucose concentration in diabetic participants (r=0.42, p=0.02) and when all the participants were pooled into one data set (r=0.30, p=0.02). There was no correlation in healthy participants (r=0.28, p=0.17). However, the authors indicated that there is significant spread in the data and concluded that sweat glucose concentration does not robustly predict that of blood. | Unclear |
| Weiner and Van Heyningen, 1952 | Lactate | Whole body sweat was collected from 2 participants using the washdown technique after a 30-min steady state stepping and a 5-min high-intensity stepping protocol in the heat (46°C, 29% rh). Blood samples were collected at rest, following steady state stepping, following high-intensity stepping, and then again following 10 min of rest. | Blood lactate concentration was significantly higher during the high-intensity protocol (12.6 mEq/L immediately after and 7.1 mEq/L 10 min after exercise) than steady state protocol (1.6 mEq/L), but sweat lactate concentration did not differ between protocols (7.7 vs. 8.2 mEq/L).  Correlation results (r values) were not reported. | No |
| Fellmann et al. 1983 | Lactate | 3 men (1 each: sedentary, fit, and very fit) completed constant load cycling (87% maximal aerobic power) and incremental cycling (from 60% to 95% of maximal aerobic power in 4-5 min increments) for 30 min each (23°C). Sweat was collected from the forehead using filter paper every 3-5 min. A blood sample was collected during each sweat collection. | During constant load cycling, blood lactate concentration did not change after the first 5 min of exercise (in the range of ~2-7 mM, depending upon fitness level, but consistent within each participant), but sweat lactate concentration decreased throughout exercise for all 3 participants (from ~15-20 mM to 11-12 mM).  During the incremental workload session, blood lactate concentrations increased (from ~3 to ~5-11mM, depending upon fitness level), but there was no change in sweat lactate concentration (~22 to 22 mM, ~18 to 16 mM, and ~14 to 12 mM in sedentary, fit, and very fit, respectively).  Correlation results (r values) were not reported. | No |
| Ament et al. 1997 | Lactate | 10 participants (9 men, 1 women) performed a ramp protocol for up to 38.5 min (325 watts). Blood samples were collected every 3.5 min. Sweat samples were collected from the subscapular region via a pouch technique at approximately the same time points as blood sampling. | As work rate increased blood lactate increased (2 to 65 mmol/L) and sweat lactate decreased (31 to 12 mmol/L). Correlation results (r values) were not reported. | Unclear |
| Green et al. 2000 | Lactate | 12 participants (6 men, 6 women) completed a 30-min constant load cycling trial and a 30-min interval cycling trial in the heat (32°C). During each trial, blood and sweat samples (from lower back via a pouch) were collected at baseline, 10 min, 20 min, and 30 min of exercise, and after 15 min rest. | Increases in blood lactate concentrations with interval cycling versus constant load cycling were not associated with a concomitant increase in sweat lactate concentration. Blood lactate concentration was significantly different between interval and constant load cycling (5.0 vs 1.5 mmol/L). Sweat lactate concentration was not significantly different between trials (~10 mmol/L) at any time point. Correlation results (r values) were not reported. | No |
| Alvear-Ordenes et al. 2005 | Lactate | Blood samples were collected from 15 amateur rugby players before, immediately post, 24-h post, 48-h post, and 72-h post-match. Sweat samples were collected before, during, 24-h post, 48-h post, and 72-h post-match from the scapular region via a gauze pad. For the pre- and post-exercise time points, sweat was collected during a Finnish sauna (81°C, 87% rh). | There was a significant increase in plasma lactate from before to immediately post-match (~1 mmol/L vs. 5.9 mmol/L), but there was no change in sweat lactate concentration (~8.8 mmol/L vs. ~8.5 mmol/L). There was no significant correlation between plasma lactate and sweat lactate concentrations (r=0.22, p>0.05). | No |
| Sakharov et al. 2010 | Lactate | 14 trained cyclists completed a stepwise incremental exercise test. Sweat was collected from the gastrocnemius at rest (via pilocarpine electrophoresis stimulation) and following exercise using a Macroduct. Venous and capillary blood samples were collected before and after exercise. | There were no significant correlations between sweat lactate concentration and capillary (r^2^=0.12, p>0.05) or venous (r^2^=0.46, p>0.05) blood lactate concentration. Authors report a significant correlation between the increase in the increment in sweat lactate and the increment in capillary blood lactate using the formula (C_lactate_ post-exercise – C_lactate_ pre-exercise) / C_lactate_ pre-exercise, to better evaluate the contribution of lactate from the working muscle independent of the lactate output from the sweat gland. Using this method the authors found a significant correlation between sweat and capillary blood (r^2^=0.72, p<0.05). | Unclear |
| Bandodkar et al. 2019 | Lactate | In 3 participants, sweat and blood were collected/analyzed 3 x per day for 1-2 days. Sweat was collected from the wrist with a novel microfluidic-based electrochemical patch. Sweat was stimulated via 15-20 min cycling exercise. | Authors concluded that there is “some potential” for using sweat to track blood lactate, but r value NA in main paper and supplemental material not available from the journal website. | Unclear |
| Czarnowski et al. 1992 | Ammonia | Sweat was collected from the ventral forearm via gauze pads after pilocarpine iontophoresis in 10 healthy control participants before and after ammonium chloride ingestion and in 4 hyperammoniemic liver failure patients. Blood samples were collected around the time of sweat collection. | Compared with control trials, ingestion of ammonium chloride led to increased plasma (37 to 54 µmol/L) and sweat (827 to 1359 µmol/L) ammonia concentrations. Liver failure patients had significantly higher baseline plasma (80 µmol/L) and sweat (1304 µmol/L) ammonia concentrations than healthy controls. Correlation results (r values) were not reported. | Unclear |
| Ament et al. 1997 | Ammonia | See Ament et al. methods above | As work rate increased blood ammonia concentration increased (~30 µmol/L to ~125 µmol/L) and sweat ammonia decreased (9.9 mmol/L to 2.8 mmol/L). Correlation results (r values) were not reported. | Unclear |
| Alvear-Ordenes et al. 2005 | Ammonia | See Alvear-Ordenes et al. methods above | There was a significant correlation between plasma and sweat ammonia concentrations (r=0.27, p<0.01). | Yes |
| Komives et al. 1966 | Urea | 4 men walked on a treadmill (46°C dry bulb, 26°C wet bulb) with turbulent air movement for 3-5 h. Whole body sweat (wash down method) and venous blood were collected at 0.5, 1.5, 2.5, 3.5, and 4.5 h. Urea was ingested (0.6 or 0.8 g/kg) just prior to the 2nd hour of exercise. | Sweat: plasma urea ratio was ~1.5 at 30 min and declined to ~1.0 at 1.5 to 4.5 h. Correlation results (r values) were not reported. | Unclear |
| al-Tamer and Hadi, 1997 | Urea | Participants were 11 women with end stage renal disease. Venous blood samples were collected immediately before dialysis. Drops of sweat were collected from the chin using a plastic disposable syringe 15 min after entering a room set at 40-45°C. | Authors reported that there was no relation between sweat and serum urea concentrations. Correlation coefficient not reported, but calculated as r=0.17 from raw data listed in paper. Sweat urea concentration was ~5.5-50x higher than serum concentration. | No |
| Huang et al. 2002 | Urea | Venous blood samples were collected from 16 men after completion of a 1 h outdoor (32°C) tennis match. Then sweat was collected from the participants back and chest via a dripping method while they sat in a hot room (40-45°C and 30-50% rh). | There was no correlation between serum and sweat urea concentrations (r=-0.07, p=0.80). Urea concentrations were higher in sweat (22.2 mmol/L) than serum (6.2 mmol/L). | No |
| Alvear-Ordenes et al. 2005 | Urea | See Alvear-Ordenes et al. methods above | There was a significant correlation between plasma and sweat urea concentrations (r=0.67, p<0.001). | Yes |
| Patterson et al. 2002 | Bicarbonate | 10 male participants completed 90-min cycling sessions at room temperature after ingestion of sodium bicarbonate (0.3 g/kg) or placebo. Sweat was collected from the scapular region via a Parafilm pouch technique during exercise. Blood was collected before ingestion, after ingestion, and after 15, 30, 60, and 90 min of exercise. | Sodium bicarbonate ingestion increased blood bicarbonate concentration and pH (p< 0.05 vs. placebo). Sweat bicarbonate concentration tended to be higher after ingestion of sodium bicarbonate compared with placebo (3.73 vs. 2.49 mEq/L, p=0.07). Sweat pH was significantly higher after sodium bicarbonate ingestion vs. placebo (6.38 vs. 6.24, p=0.01). Correlation results (r values) were not reported. | Unclear |
| Haugen and Talbert, 1928 | Amino Acids | Sweat was collected from the upper body of 30 participants using a rubber jacket technique during 15-25 min passive heating (40°C) and cycling exercise (25-30°C). Blood samples were collected immediately before and after passive heating and exercise. | Authors concluded that there were no correlations between sweat and blood amino acid concentrations for either condition or time point (r value not reported). | No |
| Hier et al. 1946 | Amino Acids | Sweat was collected from the whole body of 10 men using a rubber bag technique during 20-30 min passive heating. One participant ingested 25 g amino acids (histadine, tyrosine, isoleucine, and threonine) and blood and sweat were collected 2 h later. | Authors concluded that there were no correlations between sweat and plasma amino acid concentrations (r value not reported). With amino acid ingestion, plasma amino acid concentrations increased without a concomitant rise in sweat amino acid concentrations.  Histadine  Plasma: 20 and 555 µg/ml; Sweat: 100 and 83 µg/ml before and after ingestion, respectively  Tyrosine  Experiment 1: Plasma: 17 and 55 µg/ml; Sweat: 28 and 28 µg/ml before and after ingestion, respectively  Experiment 2: Plasma: 15 and 77 µg/ml; Sweat: 23 and 27 µg/ml before and after ingestion, respectively  Isoleucine  Plasma: 15 and 171 µg/ml; Sweat: 17 and 23 µg/ml before and after ingestion, respectively  Threonine  Plasma: 17 and 99 µg/ml; Sweat: 35 and 38 µg/ml before and after ingestion, respectively | No |
| Dunstan, 2016 | Amino Acids | Sweat was collected into a sterile jar from the back of 11 male endurance athletes immediately after three self-paced 5 km runs on a treadmill. Blood samples were collected before and after exercise. | Data from the 3 runs were averaged to provide a single representation for each participant. Participants were then ‘clustered’ into 3 groups: high (>10 mmol/L, n=4), intermediate (4-10 mmol/L, n=7), and low (<4 mmol/L, n=8) sweat total amino acid concentrations. A significant negative correlation (r=-0.99, p value NA) between clusters of sweat total amino acid concentration and resting plasma total amino acid concentration was reported. Correlation results for raw data were not reported for sweat versus pre- or post-exercise plasma amino acids. | Unclear |
| Phillips and McAloon, 1980 | Ethanol | Insensible sweat was collected from the legs of 14 participants via sweat patch technique for 2-8 days. Venous blood was collected every 2 h throughout the 8 day-period. Alcohol was consumed episodically (5 g ethanol/kg/day for only 2 days) or continually (1, 2, or 5 g ethanol/kg/day for 8 days). | There was a significant correlation between sweat and blood ethanol concentrations (r=0.93, p<0.001) | Yes |
| Buono, 1999 | Ethanol | 10 men consumed ethanol (0.8-0.9 g /kg) within 30 min. Capillary blood and forearm sweat were collected 1, 2, and 3 h following the start of ethanol ingestion. Sweat was collected with a Macroduct after pilocarpine iontophoresis. Blood was collected via capillary methods. | There was a significant correlation between sweat and blood ethanol concentrations, both before (r=0.98, slope =0.81) and after correction for water content (r=0.97, slope = 1.01). | Yes |
| Gamella et al. 2014 | Ethanol | 12 men and women ingested 30-140 ml of alcohol. Sweating was stimulated via pilocarpine iontophoresis and sweat samples were collected with a Macroduct for 30-40 min thereafter. Capillary blood collected 15 min after sweat stimulation. Ethanol concentration was measured in sweat and blood samples using gas chromatography. | There was a “good” correlation between sweat and blood ethanol concentration (r=0.91, p value NA). | Yes |
| Hauke et al. 2018 | Ethanol | 2 participants (2 trials each) ingested ~80 mL of 40% alcohol. Sweat was collected/analyzed for 3-4 h from the ventral forearm using a novel biosensor device that stimulated sweat by carbachol iontophoresis. Blood alcohol content was measured by breathalyzer every 5 min. | There was a significant correlation between sweat and blood alcohol concentration (r=0.95-0.98, p values NA). | Yes |
| Marques-Deak et al. 2006 | Cytokines | Insensible sweat was collected from the torso of 9 healthy women via two absorbent patches. Patches were applied 11:00 am and removed 24 h later. A blood sample was collected at 8:00 am the day after sweat patch application. | There were no significant differences between sweat and plasma concentrations for any cytokines. Plasma and sweat concentrations were significantly correlated for all cytokines.  IL-1α: sweat 7.6 pg/ml, plasma 5.8 pg/ml; r^2^=0.70 (p=0.005)  IL-1β: sweat 10.0 pg/ml, plasma 8.2 pg/ml; r^2^=0.79 (p=0.003)  IL-6: sweat 10.0 pg/ml, plasma 8.4 pg/ml; r^2^=0.52 (p=0.03)  TNF-α: sweat 12.8 pg/ml, plasma 9.7 pg/ml; r^2^=0.95 (p<0.0001)  IL-8: sweat 4.2 pg/ml, plasma 3.3 pg/ml; r^2^=0.81 (p=0.001)  TGF-β: sweat 3.7 pg/ml, plasma 3.2 pg/ml; r^2^=0.94 (p=0.0003) | Yes |
| Cizza et al. 2008 | Cytokines | Insensible sweat was collected from the torso of 19 women with major depressive disorder and 17 healthy control women via two absorbent patches. Patches were applied 11:00 am and removed 24 h later. A blood sample was collected at 8:00 am the day after sweat patch application. | All cytokines were significantly higher in the patient group versus controls. Plasma and sweat concentrations were significantly correlated for all cytokines in the patient group (r=0.92-0.99, p<0.001). In normal controls the correlation between plasma and sweat was significant for IL-1α (r=0.78, p=0.02), IL-1β (r=0.65, p=0.005), TNF-α (r=0.67, p=0.003), and IL-8 (r=0.63, p=0.006). The correlation for IL-6 (r=0.36, p=0.15) was not significant. | Yes |

Sweat concentration values are mean data reported in original papers.
